# Implementing a Resource-Light and Low-Code Large Language Model System for Information Extraction from Mammography Reports: A Pilot Study

**DOI:** 10.1007/s10278-025-01659-4

**Published:** 2025-09-10

**Authors:** Fabio Dennstädt, Simon Fauser, Nikola Cihoric, Max Schmerder, Paolo Lombardo, Grazia Maria Cereghetti, Sandro von Däniken, Thomas Minder, Jaro Meyer, Lawrence Chiang, Roberto Gaio, Luc Lerch, Irina Filchenko, Daniel Reichenpfader, Kerstin Denecke, Caslav Vojvodic, Igor Tatalovic, André Sander, Janna Hastings, Daniel M Aebersold, Hendrik von Tengg-Kobligk, Knud Nairz

**Affiliations:** 1 Department of Radiation Oncology, Inselspital, Bern University Hospital and University of Bern, Bern, Switzerland; 2 School of Medicine, University of St. Gallen, St. Gallen, Switzerland; 3 Department of Diagnostic, Interventional and Pediatric Radiology (DIPR), Inselspital, Bern University Hospital and University of Bern, Bern, Switzerland; 4 Department of Neurology, Inselspital, Bern University Hospital and University of Bern, Bern, Switzerland; 5 Institute for Patient-Centered Digital Health, Bern University of Applied Sciences, Biel/Bienne, Switzerland; 6 Faculty of Medicine, University of Geneva, Geneva, Switzerland; 7 Wemedoo AG, Steinhausen, Switzerland; 8 ID Berlin GmbH, Berlin, Germany; 9 Institute for Implementation Science in Health Care, University of Zurich, Zurich, Switzerland; 10 Swiss Institute of Bioinformatics, Lausanne, Switzerland

**Keywords:** Large language models, Natural language processing, Artificial intelligence, Mammography, Data extraction

## Abstract

Large language models (LLMs) have been successfully used for data extraction from free-text radiology reports. Most current studies were conducted with LLMs accessed via an application programming interface (API). We evaluated the feasibility of using open-source LLMs, deployed on limited local hardware resources for data extraction from free-text mammography reports, using a common data element (CDE)-based structure. Seventy-nine CDEs were defined by an interdisciplinary expert panel, reflecting real-world reporting practice. Sixty-one reports were classified by two independent researchers to establish ground truth. Five different open-source LLMs deployable on a single GPU were used for data extraction using the *general-classifier* Python package. Extractions were performed for five different prompt approaches with calculation of overall accuracy, micro-recall and micro-F1. Additional analyses were conducted using thresholds for the relative probability of classifications. High inter-rater agreement was observed between manual classifiers (Cohen’s kappa 0.83). Using default prompts, the LLMs achieved accuracies of 59.2–72.9%. Chain-of-thought prompting yielded mixed results, while few-shot prompting led to decreased accuracy. Adaptation of the default prompts to precisely define classification tasks improved performance for all models, with accuracies of 64.7–85.3%. Setting certainty thresholds further improved accuracies to > 90% but reduced the coverage rate to < 50%. Locally deployed open-source LLMs can effectively extract information from mammography reports, maintaining compatibility with limited computational resources. Selection and evaluation of the model and prompting strategy are critical. Clear, task-specific instructions appear crucial for high performance. Using a CDE-based framework provides clear semantics and structure for the data extraction.

**Supplementary Information:**

The online version contains supplementary material available at 10.1007/s10278-025-01659-4.

## Introduction

### Synoptic Reporting and Automated Extraction of Data from Radiological Documents

Synoptic or structured reporting is an approach to providing a structured overview of a radiological report for a given radiological examination. Synoptic reporting has numerous advantages over free-text reporting, including improved consistency, clarity, and completeness of information [1]. Despite these advantages, synoptic reporting is not widely adopted in radiology [2] due to the increased time requirements, technological challenges, as well as the lack of standardization [3, 4].

Automated extraction of information from radiological reports is therefore of longstanding interest, as it could automatically generate corresponding synoptic reports in a standardized and precise way from free-text radiological documents [5]. Classical natural language processing (NLP) techniques for text classification, like bag-of-words models or term-frequency-inverse document frequency (TF-IDF), have achieved success in extracting standardized data (e.g., names, dates, etc.) [6]. However, for more complex relevant information in radiological reports (e.g., peculiarities regarding certain morphological aspects, etc.), the performance of such classical NLP approaches has been limited [7]. (Small) language models such as BERT, when given training for specific extraction tasks, can perform very well, albeit not in a general multi-purpose instruction-following way [8, 9].

Recent advances in large language models (LLMs) have opened new opportunities for the automation of tasks that traditionally require human-level context understanding and reasoning. LLMs have been shown to be useful in structuring radiology reports [10], as well as in other medical settings, including writing clinical letters [11], clinical decision-support [12], medical education [13], or screening medical literature [14].

Furthermore, LLMs have been shown to be highly effective for extracting information from unstructured clinical documents, substantially facilitating the automated generation of synoptic reports [15]. Several studies have demonstrated successful solutions using proprietary LLMs provided by private companies, such as OpenAI’s GPT-4 [16] or Anthropic’s Claude 3.5 [17], which are accessible via application programming interfaces (APIs). While such models demonstrate powerful performance, they rely on external data transmission, thus raising critical concerns about clinical applications, particularly when dealing with sensitive patient data. To comply with data privacy regulations and prevent the transmission of sensitive information to external stakeholders, LLMs used within healthcare institutions should ideally operate on local hardware [18].

Therefore, the research for practical implementation is now focusing on locally deployed open-source LLMs for the extraction of data from clinical documents [19]. However, many of the most capable LLMs are very, large and for several tasks a correlation between model size and performance has been reported [20]. Many powerful models thus demand substantial computational resources and advanced hardware infrastructure to operate efficiently [21]. This highlights the importance of balancing technical feasibility with practical considerations in clinical applications [22].

### Clear Data Definitions Using the Common Data Element (CDE) Concept

Regardless of the NLP technique used for data extraction, clearly defined data concepts with precise meanings are crucial. The National Institutes of Health (NIH) introduced the concept of common data elements (CDEs) in 2011. As stated in the definition of the NIH, a “*Common data element (CDE) is a standardized, precisely defined question, paired with a set of allowable responses, used systematically across different sites, studies, or clinical trials to ensure consistent data collection*” [23]. Initially designed for clinical trials, CDEs are increasingly applied for the collection of real-world data (RWD) [24].

Moreover, CDEs facilitate seamless implementation in information technology systems through standardized data formats such as JSON or XML, making them particularly relevant in the era of big data and machine learning [23].

For these reasons, CDEs were chosen as the fundamental semantic concept for the collection and management of RWD within the SMARAGD (Smart Radiology Goes Digital) initiative [25]. SMARAGD is a collaborative effort between academia and industry, that leverages state-of-the-art NLP techniques to extract and manage clinical data, with a particular focus on the care pathway of breast cancer patients undergoing radiological examinations.

### Practical Implementation of LLMs for Automated Information Retrieval from Mammography Reports in a Local Setting

As part of the SMARAGD initiative, this pilot study focuses on the practical realization of an LLM-based system for extracting information from free-text mammography reports. By using the modular and interoperable framework provided by CDEs, this system has been integrated into a broader data management architecture, demonstrating the potential of advanced NLP techniques in enhancing clinical workflows. Mammography reports are a critical and frequent form of medical report. While these reports often adhere to semi-structured guidelines like BI-RADS, they seldom use synoptic reporting, which provides a more structured, checklist-style format [26]. Practical, automated data extraction (and thereby translation of free-text into synoptic reports) would be highly valuable for facilitating potential downstream applications (such as general data analysis, error detection, or quality assurance).

The primary objective of this work was to create a practical, easy-to-deploy, and cost-effective system for extracting categorical CDE values from free-text radiology reports. This system ensures that all data is retained within the local environment and can be deployed on local hardware with limited computational resources. By leveraging open-source LLMs, the system eliminates external dependencies, effectively addressing data privacy concerns while also meeting the need for affordability in clinical applications.

## Methods

A schematic overview of the methodology used in the study is provided in Supplementary Fig. 1.

### Development of CDE Resources and Data Structure

As the first step, an interdisciplinary expert panel comprising physicians, healthcare informaticians, and computer scientists developed resources for generating synoptic reports with categorical (“value list”) CDEs suitable for the clinical environment based on published NIH guidelines [23, 27].

The focus of CDE development was to ensure alignment with real-world clinical documentation and established reporting standards, specifically the guidelines of the American College of Radiology (ACR) on mammography reporting [28]. Based on previous research for defining CDEs in real-world clinical documents [29], the panel created and iteratively refined CDE resources until consensus was reached among the involved experts.

As the second step, CDEs were further evaluated by radiologists who used them in clinical practice for local documentation. Their feedback informed iterative revisions and the finalization of the data concepts.

As the third step, data elements were organized into a hierarchical structure [30], consistent with the NIH’s CDE framework (corresponding to CDE forms) [31].

### LLM-Based Data Extraction System in a Local Setting

#### General Approach

As previously shown, LLMs can be used for effective data extraction from clinical documents [32]. For our use case, the LLM was implemented as a basic data extraction and classification system as part of a broader framework, based on previous studies [14, 33]. We used the recently published *general-classifier* Python package [34], which allows for easy-to-use deployment of LLMs for text classification. It facilitates the classification of a text (i.e., in our case the mammography report) into user-defined topics with mutually exclusive categories.

The Python package has been used for biomedical literature classification with high levels of accuracy, with details on the functionality reported previously [33]. In brief, the library provides an easy-to-use interface for defining structured templates within which an LLM is executed; for each template, the LLM is provided with a prompt containing an instruction and the text from the medical document and is asked to answer a question about this text. This structure can be saved as a machine-processable JSON file and later imported to perform a classification. In general, LLMs function by predicting how a text (being a sequence of minimal text segments, so-called tokens) is likely to be continued. The probability of the next token in a given token sequence is calculated using a neural network and is based on the data used during model training. The probability of the next tokens associated with the value of given categories for a classification task can be calculated. The LLM can be used in this way as a general classification system by selecting the category with the highest probability.

**Fig. 1:**
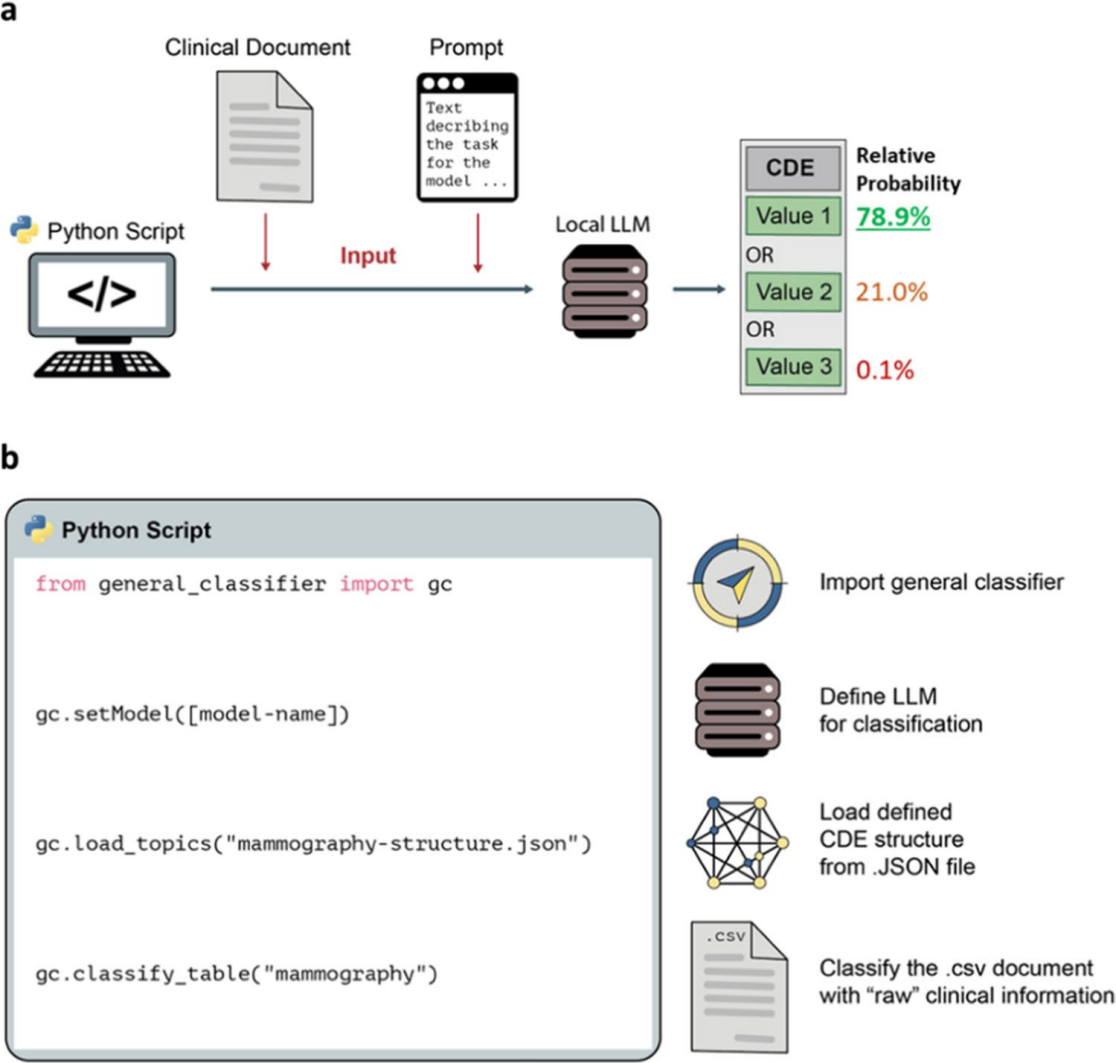
**a **Schematic illustration of the LLM-based approach for extracting the value of a categorical “Value List” CDE from a clinical document using LLMs. **b **Principle of using the *general-classifier* framework for the data extraction. With three lines of code an LLM is initialized, the data structure (in .JSON) is loaded and the clinical documents (in .CSV) are analyzed accordingly

Figure 1a illustrates the approach for a “value list” CDE with three possible values (categories) from which the LLM has to select the correct value. For each of the values, the probability is calculated and the value with the highest relative probability is selected. An example of such a classification task might be the CDE “laterality of known breast cancer” with the possible values/answers “left,” “right,” and “unknown (no information provided in the document).”

It should be noted that the purpose of this system was to create a pipeline for data extraction by answering clearly defined questions based on a given report, which had been created by a physician. It thereby translates information already given in the report into a structured format. The purpose was not to create a system that independently infers new information (like, e.g., assigning a BI-RADS score to a given report).

#### Script and Hardware

A simple Python script was developed to implement the approach (Fig. 1b; available on GitHub [35]). Based on the data structure for the reports, a corresponding JSON structure containing the classification topics, categories, and hierarchies was created for use with the *general-classifier* Python package (version 0.1.10).

The system was deployed on a local Linux server with an RTX6000 Nvidia GPU (with 48GB RAM) to ensure optimal processing capacity for running an open-source LLM. The software environment comprised Python (version 3.11.5) and PyTorch (version 2.4.0) [36], alongside dependencies specific to the open-source LLMs used.

#### Models

Five different modern open-source LLMs were used in the study. The LLMs were selected because of reported high performance on different relevant benchmarks such as MMLU [37], IFEval [38], and GPQA [39].

- Rombos-LLM-V2.6-Qwen-14b (Rombos) is a fine-tuned version of Alibaba’s Qwen2.5-14B model for precise instruction following [40]. The Qwen series excels in language understanding, generation, multilingual tasks, coding, math, and reasoning [35]. It has 14.8 billion parameters and a size of 29.57 GB.
- SOLAR Pro Preview Instruct (solar preview) is an instruction-tuned LLM by Upstage, reported to be the *“most intelligent LLM on a single GPU”* [41]*.* It is a preview version of the SOLAR Pro model, optimized for following user instructions and generating helpful responses. It has 22.1 billion parameters and a size of 44.43 GB.
- Phi-4 is a compact AI language model developed by Microsoft, released in February 2025 [42]. It delivers strong performance on reasoning, coding, and math tasks despite its small size. Trained on high-quality data, it offers capabilities comparable to much larger models while requiring fewer computational resources. It has 14.7 billion parameters and a size of 29.34 GB.
- Li-14B-v0.4 (Li) is an LLM developed by the Chinese company Century Innovation [43]. Like the Rombos model, it is a fine-tuned version of the Qwen2.5-14B model. It strikes a balance between computational efficiency and performance capabilities. At the time of the study (as of February 2025), it was ranked first among LLMs up to 15B parameters on the Open LLM Leaderboard provided by Huggingface [44]. It has 14.8 billion parameters and a size of 29.52 GB.
- Lamarck 14B v0.7 (Lamarck) is a high-performing language model optimized for limited execution on hardware with limited resources [45]. Created through sophisticated merging techniques, it combines influences from multiple top models, including Virtuoso-Small [46], DeepSeek [47], and DRT-o1 [48]. The model excels in multi-step reasoning, prose generation, and multilingual capabilities, using a custom toolchain of Low-Rank Adaptations (LoRAs) and targeted layer merges to achieve its balanced performance profile. It has 14.8 billion parameters and a size of 29.51 GB.

The selection of the five open-source LLMs for this study reflects the diverse approaches to building powerful and efficient language models. Each model has unique characteristics. For instance, Rombos and Li demonstrate how the same base model can be adapted differently through specialized fine-tuning versus performance-broadening model merging. In contrast, SOLAR utilizes a unique “depth up-scaling” architecture to maximize performance on limited hardware. Phi-4’s strength stems from its training on high-quality synthetic and curated data, while Lamarck represents a creative “model cocktail” approach, combining several distinct models to create a versatile and well-rounded system. This variety showcases the different strategies employed to balance performance, efficiency, and task specialization.

#### Prompting

As the LLM-based data extraction system depends on the output of the LLM, it also depends on the input prompt given to the model. As shown, the performance of LLMs on various tasks can be substantially increased by optimizing the prompt, a so-called prompt engineering technique [49, 50]. In a first evaluation, the default prompt for classification tasks provided in the *general-classifier* package was used. This default prompt contains a general instruction to conduct a classification with the name of the topic and the possible categories; see also [34].

In additional evaluation runs, the established prompting techniques of few-shot prompting ([51]; 1-shot and 3-shot) and chain-of-thought (CoT) prompting [52] were used within the framework. Furthermore, an additional evaluation run was conducted after manual adaptation of the default prompt, aiming to precisely define the classification task for the individual CDEs.

These adapted prompts were systematically created, mimicking a question–answer dialog using the following structure:

- INSTRUCTION – containing information that the task is to answer a specific question based on the information provided in a German-language mammography report.
- QUESTION – containing the defined question that is to be answered.
- TEXT – the text of the mammography report; based on the *general-classifier* Python package, the string “[TEXT]” is inserted, which will be replaced with the given text for the classification.
- ANSWER – starting the text answer which would be given to the question. This text ends the prompt and provides a direct context for the classification.

The JSON files containing the default, adapted, and CoT prompts used in the study are available on GitHub [35].

### Dataset and Manual Value Assignment

To construct the dataset for this study, 61 anonymized mammography reports were utilized. These reports were from patients who had been referred for breast diagnostics of potential tumor lesions. The mammography reports are free-text reports following basic reporting recommendations of BI-RADS. In the dataset, a total of 34 lesions were mentioned for 25 of the patients (41.0%). Sixteen patients (26.2%) already had a diagnosis of previous breast cancer in the medical history. For both breasts, about two-thirds of the reports assessed the likelihood of breast cancer as very low (BI-RADS 1 or 2). About a third of both breasts were scored B and C (based on the scoring system of the American College of Radiology [ACR]), respectively. Further details are presented in Supplementary Table 1. All patients had filled out a general consent form for the secondary use of their medical information for scientific studies. Apart from that, there were no other criteria for inclusion or exclusion. Based on those mammography reports and our CDE-based template, the task of obtaining the corresponding “correct” CDE values was assigned to two physicians independently. Both physicians were residents (three and one years of clinical experience), who received training in mammography reporting and instructions for the value assignment. In case of disagreement between the two clinicians, a final decision was made by the study coordinators. A final ground truth dataset with a total of 1533 CDE values assigned to the 61 reports was created. The conduction of the study was approved by the local ethics committee (BASEC Project ID 2022–01621).

### Statistical Analysis

The inter-rater agreement (IRA) between the two researchers was calculated using Cohen’s kappa [53], which is a standard metric to measure reliability for categorical ratings while accounting for agreement that occurs by chance.

To evaluate the performance of the LLMs, we calculated several key metrics. The primary metric was overall accuracy, also known as the exact-match proportion. This metric calculates the percentage of all classification tasks across all reports that the LLM answered correctly. Overall accuracy provides a straightforward and easily interpretable measure of the system’s performance at a high level [54]. We additionally calculated both the micro-averaged and macro-averaged recall, precision, and F1-Score. These metrics are adequate for this study due to the nature of the dataset with a hierarchy of classification tasks [54]. Because of this, some CDEs are only applicable if a “parent CDE” has a specific value. For instance, questions about the description of a tumor lesion are only relevant if a tumor lesion is indeed mentioned in a report. This creates a scenario where the number of instances for each CDE can be highly variable, leading to potential class imbalance. Micro-averaging calculates metrics by looking at all individual predictions combined. This method gives more weight to larger classes, making it a good measure of overall performance across the entire dataset. Macro-averaging calculates metrics for each class independently and then takes their average. This treats all classes as equally important, regardless of their size, which is useful for understanding how the model performs on average for each category, including rare ones.

To assess statistically significant differences, we compared the performance of all five LLMs in a pairwise manner. We used a paired bootstrap resampling procedure with 2000 iterations as an adequate non-parametric test for comparison on the same dataset [55]. For each pair of LLMs, we calculated the difference in performance across overall accuracy, micro- and macro-averaged recall, precision, and F1-score. All metrics were computed on a cell-by-cell basis, excluding data points where no ground truth was available. Statistical analysis was conducted in Python using the pandas (version 2.3.1) [56], numpy (version 2.3.1) [57] and scikit-learn (version 1.7.0) [58] libraries. To account for the multiple comparisons problem (5 × 2 = 10 pairs per metric), we applied a Bonferroni correction [59]. A difference was considered statistically significant only if its two-sided bootstrap *p*-value was less than the corrected threshold of *p* < 0.005.

In an additional sub-analysis, the overall accuracies for the default and the adapted prompt were calculated only for the cases for which the LLM provided a high relative probability for the selected category (= probability for the selected category divided by the sum of probabilities for all categories). A threshold for this parameter reflects the *“certainty of the model”* when performing the classification. Additional sub-analyses were conducted for the thresholds of 90%, 99%, and 99.9%. Apart from the overall accuracies, the proportion of classifications above the thresholds (coverage rate) was calculated.

### Time Measurement and Cost Estimation of Used Hardware

The required time for the data extraction was calculated using the developed Python script [35]. A basic cost calculation of the used hardware infrastructure was conducted. A basic cost estimation for the used components was conducted in Swiss Francs, with conversions into euros and US dollars based on the exchange rates from January 16, 2025.

## Results

### CDE-Based Data Structure for the Presentation of the Mammography Reports

**Fig. 2:**
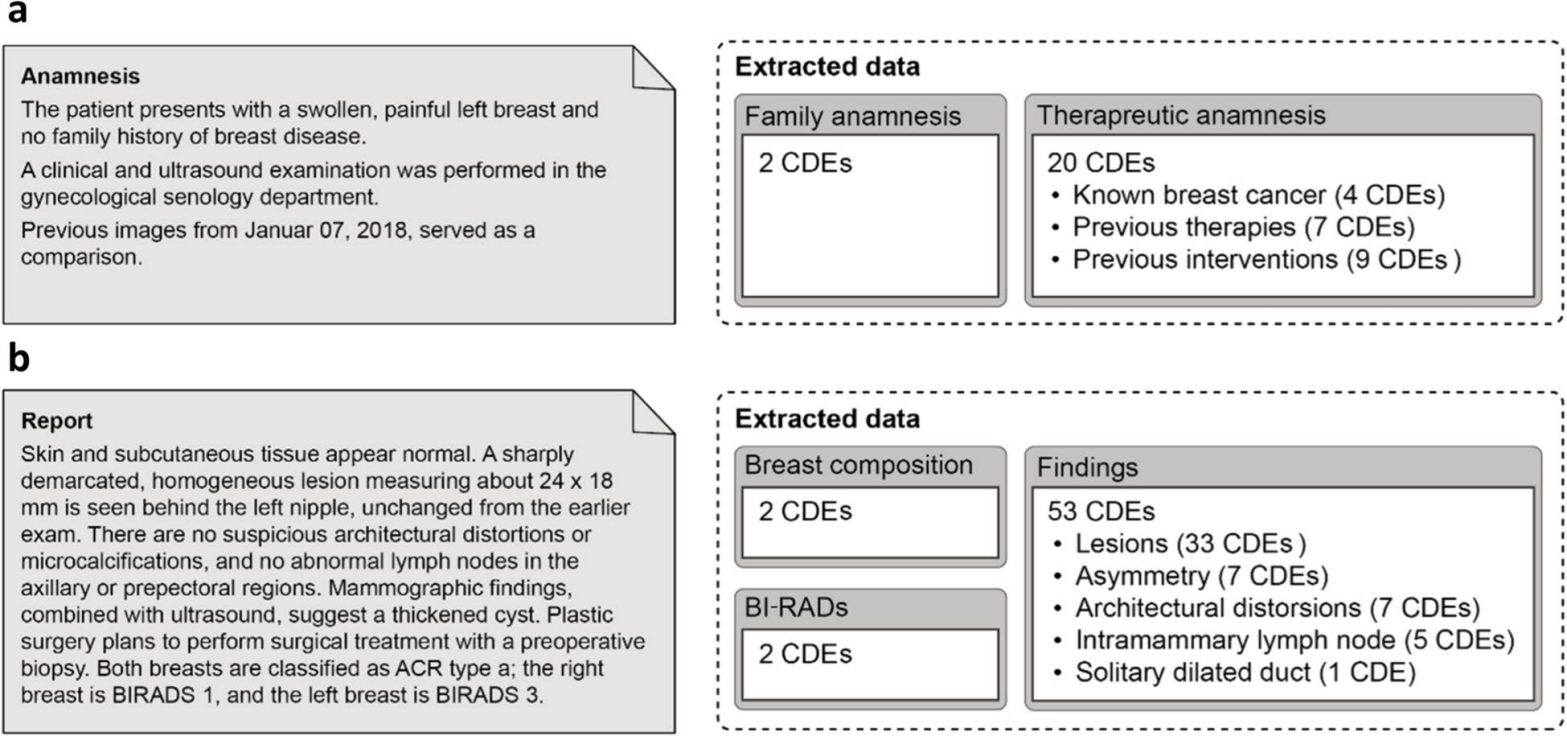
Basic hierarchical structure for the data extracted from the mammography reports. Grouping of CDEs with the number of CDEs per group and subgroup

The finalized CDE-based data structure consisted of 79 CDEs organized into two main groups and five sub-groups. The structure of the analyzed mammography reports included a pre-text (anamnesis) that outlines the patient’s history and reason for admission, followed by the main report text. Consequently, the two main groups of the data structure were defined as “Anamnesis,” with the sub-groups “Family anamnesis” and “Therapeutic anamnesis,” and “Report,” with the sub-groups “Breast composition,” “BI-RADS,” and “Findings.” A schematic overview of the data structure with a translated artificial sample of a mammography report is provided in Fig. 2.

A detailed list of CDEs and data structure is provided in Appendix 1

### Interrater Agreement

A high level of IRA with a Cohen’s Kappa of 0.83 was observed between the two researchers performing the manual value assignment. The smallest level of agreement was seen for the group “Findings” with a value of 0.71. An overview of the values for the individual groups is provided in Table 1. Detailed results on all individual CDEs are provided in Supplementary Table 2.

**Table 1:** Inter-rater agreement on the different (sub-)groups of CDEs between the two independent researchers assigning the values

		Cohen’s Kappa	n
**Anamnesis**		0.90	544
	Family anamnesis	0.94	74
	Therapeutic anamnesis	0.90	470
**Report**		0.80	989
	Breast composition	0.92	122
	Findings	0.71	745
	BIRADS	0.95	122
**Overall**		0.83	1533

### Performance of the LLM-Based Data Extraction with Default Prompts

Using the default classification prompt of the *general-classifier* Python package (See also [34]) for all CDEs, the system achieved an overall accuracy of 72.9% [95% CI 69.5–76.4%] for Rombos, 61.6% [95% CI 58.5–64.9%] for solar preview, 59.2% [95% CI 56.0–62.6%] for Phi-4, 64.7% [95% CI 61.1–68.7%] for Li, and 68.5% [95% CI 65.2–72.2%] for Lamarck. The overall accuracy of Rombos was significantly higher compared to each of the other LLMs (*p* < 0.0001; see also Supplementary Table 4).

**Fig. 3:**
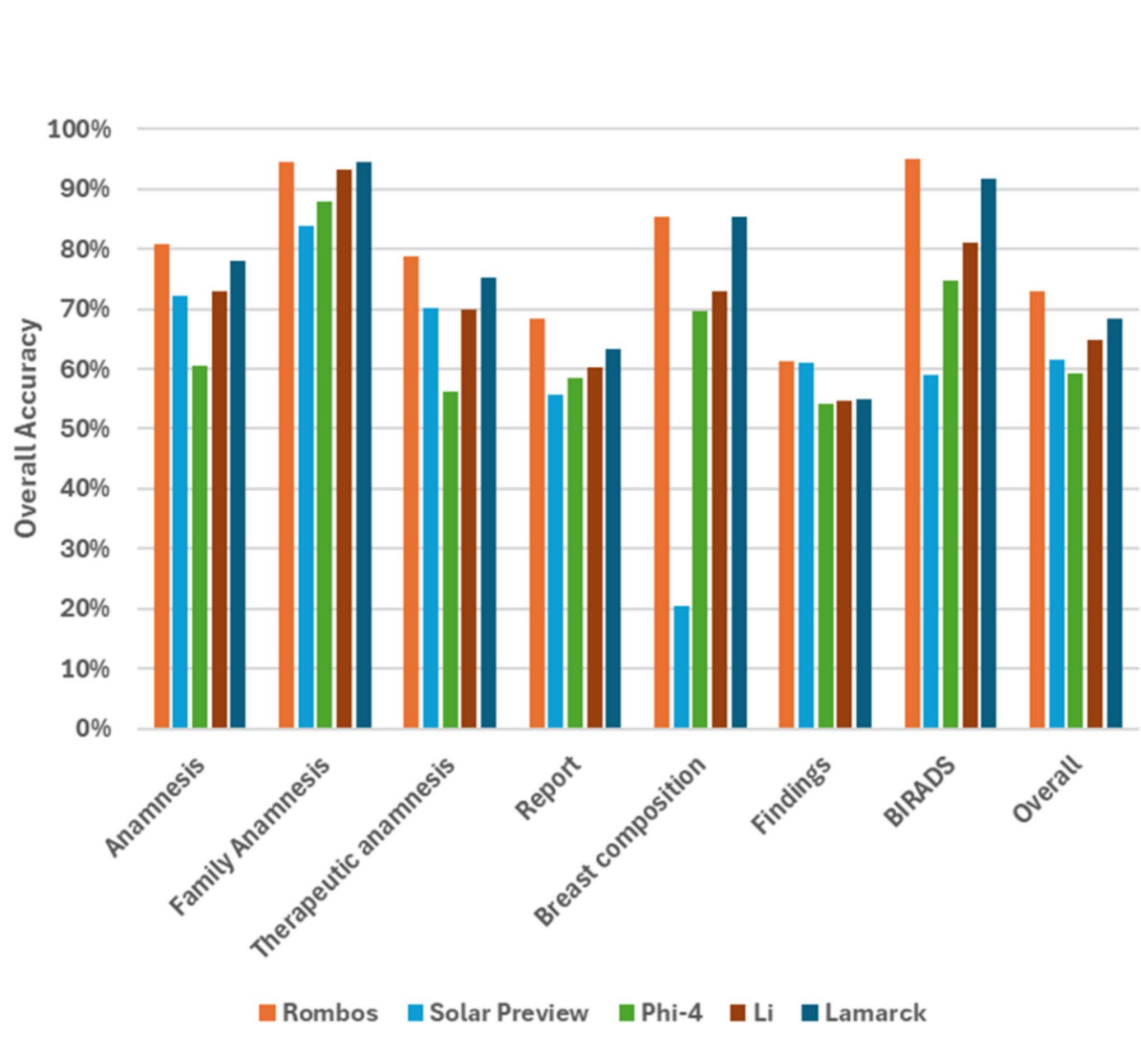
Overall accuracy of the LLM-based classification system with the five LLMs on the different groups, subgroups, and overall, in classifying the mammography reports. Results for the default prompt

Further details with results on the individual groups, sub-groups, and individual CDEs are presented in Fig. 3 and Supplementary Table 3. The results of micro- and macro-averaged recall, precision, and F1-score are provided in Supplementary Figs. 2, 3, 4, 5, 6, and 7 and Supplementary Tables 5, 6, 7, 8, 9, and 10.

### Performance of the LLM-Based Data Extraction with Different Prompting Techniques

Using the CoT prompts increased the overall accuracy for Rombos to 76.3% [+ 3.4%; 95% CI 73.3–79.5%], for Li-14 to 65.9% [+ 1.2%; 95% CI 63.7–68.9%], and for Lamarck to 72.1% [+ 3.5%; 95% CI 69.3–75.9%], while it decreased the accuracies for solar preview to 55.3% [− 6.3%; 95% CI 51.7–59.1%] and for Phi-4 to 49.8% [− 9.5%; 95% CI 46.4–53.0%].

Few-shot prompting (both 1- and 3-shot prompts) decreased the overall accuracy for all models, with a range of 30.9–67.5% for the 1-shot prompt and 32.3–58.5% for the 3-shot prompt (see Table 2 for detailed results).

**Table 2:** Overall accuracies of the five LLMs using different prompting techniques for data extraction

	Default prompt	1-Shot	3-Shot	CoT	Adapted prompt
Rombos	72.9 [69.5–76.4]	67.5 [64.3–71.3]	57.1 [55.2–59.3]	76.3 [73.3–79.5]	85.3 [82.5–88.0]
Solar preview	61.6 [58.5–64.9]	45.3 [41.9–48.8]	58.5 [56.0–61.2]	55.3 [51.7–59.1]	64.7 [62.1–67.7]
Phi-4	59.2 [56.0–62.6]	30.9 [28.8–32.9]	32.3 [29.5–35.0]	49.8 [46.4–53.0]	65.0 [62.1–68.0]
Li	64.7 [61.1–68.7]	64.3 [61.1–68.1]	45.2 [42.1–48.3]	65.9 [63.7–68.9]	77.4 [74.2–81.1]
Lamarck	68.5 [65.2–72.2]	65.0 [62.1–68.2]	55.4 [53.0–58.0]	72.1 [69.3–75.9]	81.6 [78.9–84.6]

*CoT*, chain of thought

Values in %. Values in brackets represent the 95% confidence interval based on the bootstrap resampling method

**Fig. 4:**
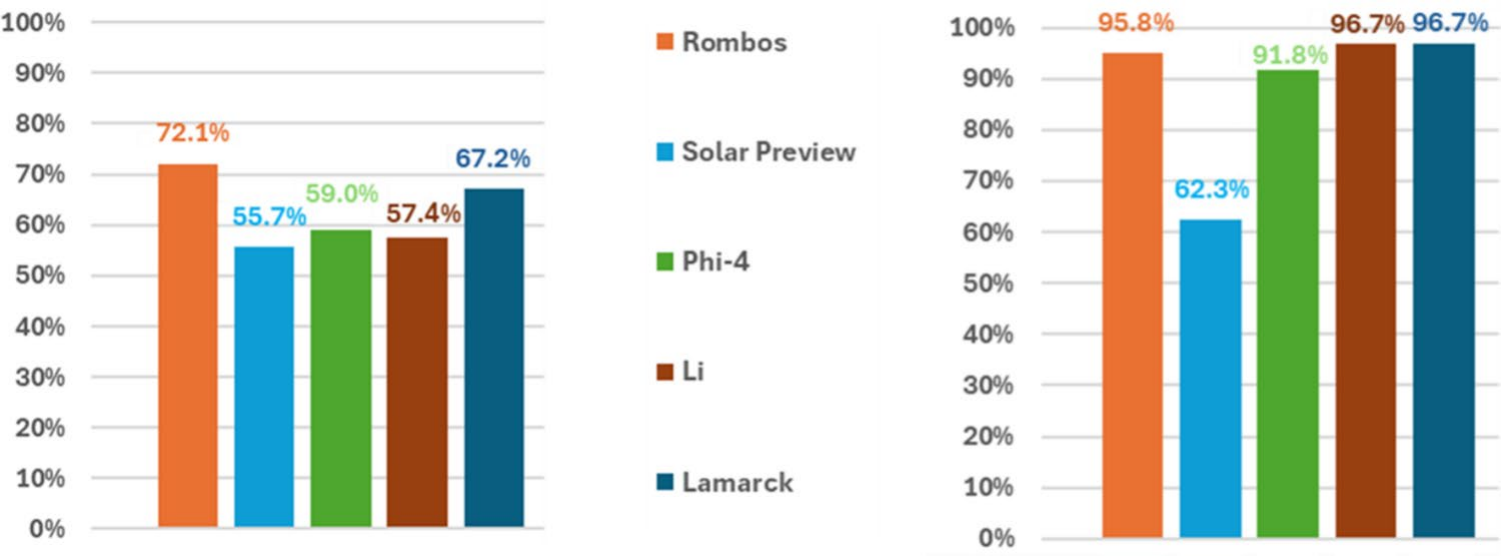
Comparison of the default prompt provided by the general classifier and the adapted prompt, better explaining the classification task for the CDE “Previously conducted mammography mentioned?”. Comparisons of accuracies for the different LLMs. The general classifier replaces [TOPIC] with the classification topic “Zuvor erfolgte Mammographie erwähnt?” (= “Previously conducted mammography mentioned?”) and [CATEGORIES] with the classification categories “ja, nein” (= “yes, no”)

Compared to the default prompt, adaptation of the prompts to clearly explain the classification tasks improved the overall accuracies for all models. A comparison between default and adapted prompt for the CDE “Previously conducted mammography mentioned?” is provided in Fig. 4 (results of the statistical analysis in Supplementary Table 11), for which accuracies increased from 55.7–72.1% to 62.3–96.7%. Using the adapted prompts, the system reached overall accuracies of 85.3% [95% CI 82.5–88.0%] for Rombos, of 64.7% [95% CI 62.1–67.7%] for solar preview, of 65.0% [95% CI 62.1–68.0%] for Phi-4, 77.4% [95% CI 74.2–81.1%] for Li, and 81.6% [95% CI 78.9–84.6%] for Lamarck. Detailed results are presented in Supplementary Figs. 8, 9, 10, 11, 12, and 13.

An overview of the overall accuracies using the different prompting techniques for the five LLMs is provided in Table 2.

### Sub-Analysis with Threshold for Relative Probability

In the sub-analysis using a threshold for the relative probability (= *“certainty”*), an increase in accuracy compared to the baseline was seen across all models and thresholds.

Using the default prompt, compared to baseline, focusing only on the cases with > 99.9% relative probability led to 86.5% accuracy (+ 13.6%; coverage rate of 44.0%) for Rombos, 94.4% for solar preview (+ 29.0%; coverage rate of 10.4%), 80.0% for Phi-4 (+ 32.5%; coverage rate of 2.0%), 72.7% (+ 7.9%; coverage rate of 16.0%) and 79.9% for Lamarck (+ 11.4%; coverage rate of 37.9%).

Similar improvements were observed when using the adapted prompts, with accuracies of 94.5% (+ 9.2%; 44.0% coverage rate) for Rombos, 93.8% for solar preview (+ 29.0%; coverage rate of 12.5%), 97.5% for phi-4 (+ 32.5%; coverage rate of 8.0%), 92.4% for Li (+ 15.0%; coverage rate of 20.7%) and 91.7% for Lamarck (+ 10.1%; coverage rate of 39.4%).

**Fig. 5:**
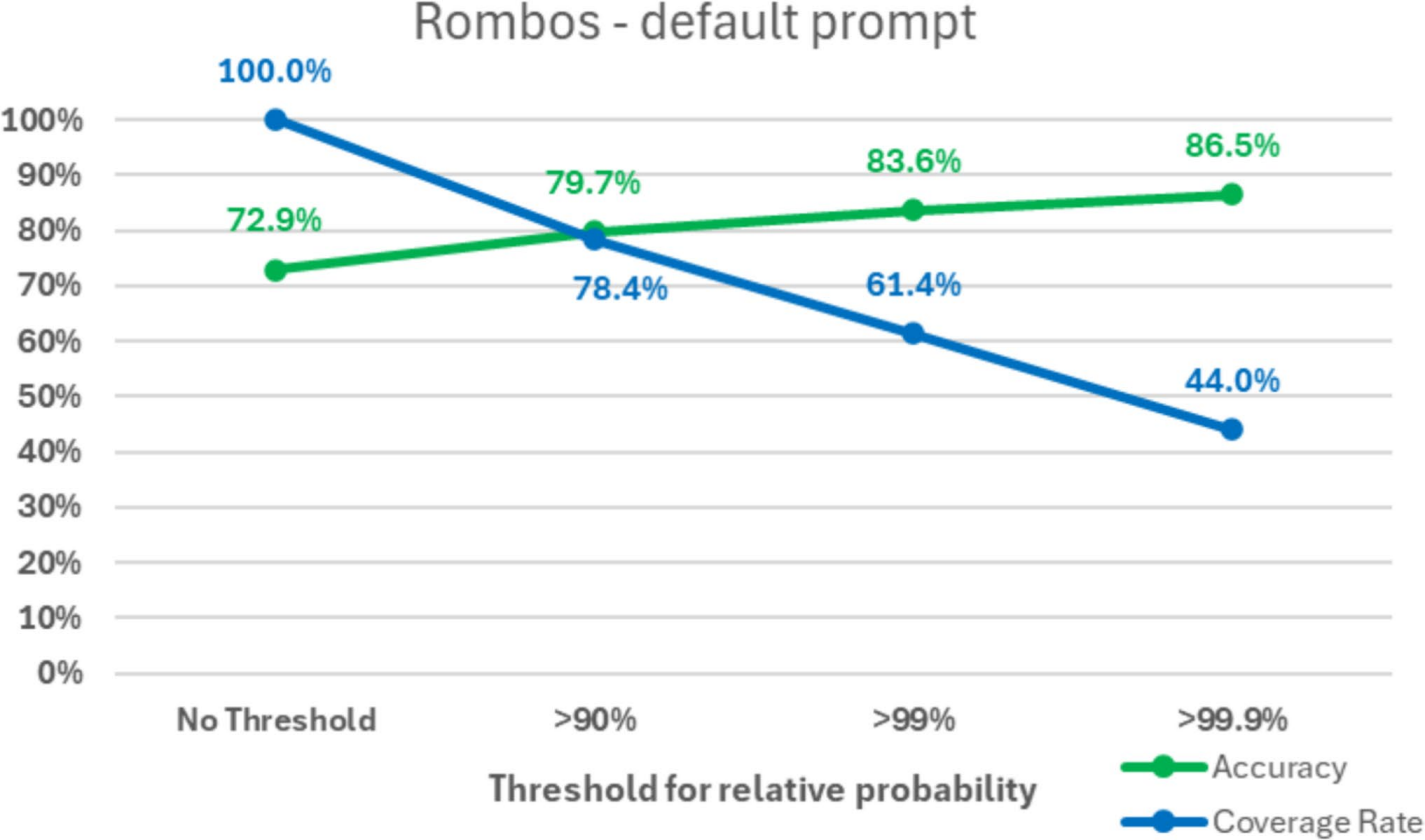
Rate of accuracy and coverage for the classifications using the default prompt with the Rombos model, depending on the threshold for the relative probability

**Fig. 6:**
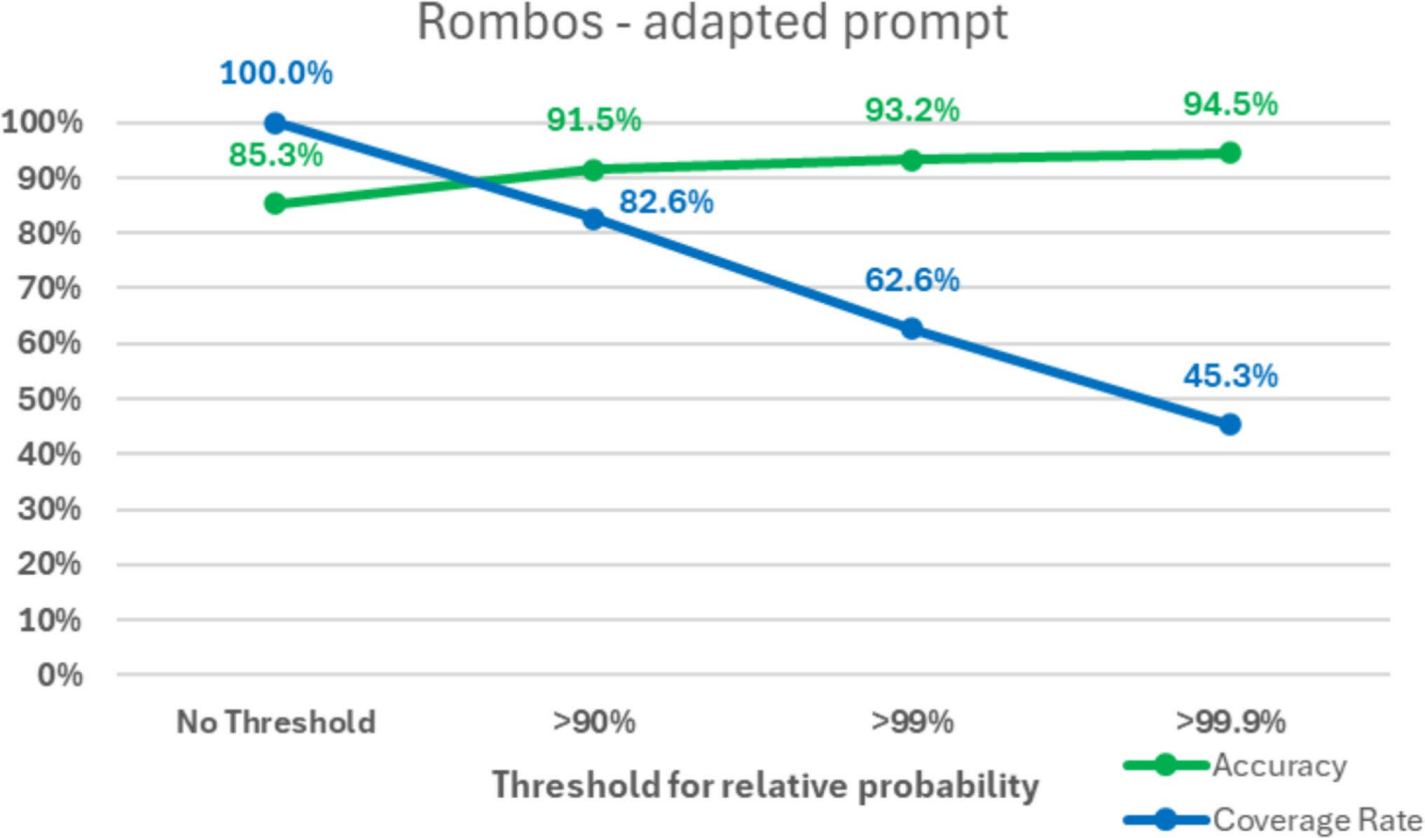
Rate of accuracy and coverage for the classifications using the adapted prompt with the Rombos model, depending on the threshold for the relative probability

The results are presented in Fig. 5 (default prompt) and Fig. 6 (adapted prompt) for Rombos, as well as in Supplementary Figs. 14, 15, 16, and 17 for the other LLMs.

### Time and Costs

For the default prompt, the script was executed in 2665.7 s for Rombos (43.7 s/report), in 6581.7 s for solar preview (107.9 s/report), 2489.5 s for Phi-4 (40.8 s/report), 2625.3 s for Li (43.0 s/report) and 2746.6 s for Lamarck (45.0 s/report). The results for the other prompts are presented in Supplementary Table 12.

The overall hardware costs of the used components were estimated at 7600 CHF, with details provided in Table 3.

**Table 3:** Basic cost estimation of the used hardware components

	**Costs in CHF**
**Core components**	
CPU: Intel Core i7-13700K	≈ 350
Motherboard: Z790	≈ 250
RAM: 64 GB DDR5	≈ 320
Storage: 1 TB NVMe SSD	≈ 120
GPU: NVIDIA RTX 6000 (Ada) With 48GB on-board RAM	≈ 6000
PSU: 1000W	≈ 190
Case/Chassis:	≈ 100
Cooling	≈ 80
**Peripherals**	
Monitor	≈ 150
Keyboard and mouse	≈ 40
** Total**	≈ 7600 (ca. 8100 € or 8300 USD)

## Discussion

### Automated Data Extraction Using Different NLP Techniques

Due to the recent advancements in the performance of LLMs, these models can now successfully perform various clinical tasks that for humans would require reasoning and contextual understanding. Various groups have demonstrated successful extraction of clinical data from mammography, CT, and MRI reports [32, 60, 61]. Many of these studies have used closed LLMs only accessible via API, such as OpenAI’s GPT models. While API-based models provide a ready-to-use and affordable opportunity to implement LLMs, such systems rely on external stakeholders, and (at least anonymized) data has to be sent outside of the local data environment. To avoid external transmission of data, research interest has grown in using locally deployed LLMs for data extraction [62, 63].

In a recent study, Woźnicki et al. used the open-source LLM Llama-2-70B-chat to extract data from chest radiograph reports [19]. They achieved an F1 score of 0.70 for English and 0.68 for German reports and, similarly to our study, noticed considerable variations in performance and understanding of semantics depending on the data to be extracted. Obviously, these results are not comparable, since different data fields were extracted and different datasets were used. However, the fact that we were able to achieve similarly high accuracies with LLMs having 1/5–1/3 of the parameters aligns with the overall trend in LLM research, with models rapidly becoming more efficient and more powerful [64]. While the work of Woźnicki et al. was published just a few months ago, Llama-2-70B-chat is not considered state-of-the-art anymore, with other open-source models like DeepSeek-R1 [47] released, demonstrating the rapid pace of development in the field.

Overall, as noted in recent reviews such as that of Pereira et al. [57], automated labeling and NLP for radiology reports have evolved from rule-based systems to highly specialized models. For instance, a work by Bozkurt et al. from 2016 [65] demonstrated the feasibility of automated information extraction from mammography reports using a rule-based NLP system. They directly linked their system to a separate Bayesian decision-support model, demonstrating the direct application value of structured data extraction. In another recent study, Dehghani et al. used a BERT-based model, MammoBERT, for the efficient labeling of French mammography reports [66]. Their approach focused on the specific task of identifying prior surgeries and their laterality. To achieve this, they employed a hybrid methodology, initially fine-tuning their model on a small dataset annotated by radiologists and then expanding the training data using a rule-based system and an active learning loop. This data-centric approach of extensive fine-tuning and data augmentation on a narrowly defined task allowed them to achieve an accuracy of 82% on unseen data.

In contrast, our study utilizes general-purpose LLMs for a much broader task involving the extraction of 79 distinct data elements. Our methodology is therefore model-interaction-centric, relying on prompt engineering for flexibility rather than data-centric fine-tuning. This highlights a complementary research direction focused on broad applicability and ease of deployment. Compared to classical NLP techniques, querying LLMs for data extraction requires more computational resources and sufficient hardware. On the other hand, traditional techniques are reliant on task-specific training or optimization. Both approaches therefore have distinct advantages and disadvantages.

As part of the SMARAGD initiative, we developed an LLM-based data extraction system using an open-source model executed on local hardware. We also focused on the practical implementation, considering environments with limited hardware resources and addressing data privacy issues by keeping medical data within a local environment.

### Performance and Practical Implementation

Even without any optimization, an accuracy of > 70% in answering medical questions about a report was achieved in our study. However, the system failed to consistently give the correct answers. Since a perfect system with 100% accuracy will not be possible, it is therefore important to mitigate the risks and better understand the situations in which there is a high risk of incorrect classifications. Different prompting techniques, such as few-shot prompting [51] or CoT prompting [52], have been proposed as possible strategies to improve LLM performance in some tasks. While the CoT prompts in our study indeed improved performance in some situations, few-shot prompting (1- and 3-shot) led to worse results. Worse performance of LLMs using few-shot prompts has been reported previously, as it is not in all cases a robust strategy [67]. Particularly for “smaller” LLMs and long few-shot prompts (each “shot” includes an entire mammography report), such as those used in our study, few-shot prompting has been reported to impair LLM output [68]. In such situations, additional, text-heavy samples lead to information overload and “confusion” for an LLM, which consequently leads to worse performance. Clear definitions for the classification tasks (as used in the adapted prompts) appear to be crucial for good performance [69, 70]. This is not surprising per se, as less clear definitions and tasks are generally more difficult to answer (also for humans).

The possibility to calculate the “relative probability” for a category, resembling the “certainty” of an LLM when doing a classification, is also very valuable. As we have seen, setting a threshold with high certainty of 90%, 99%, or 99.9% can indeed be used to achieve higher performance. Depending on how critical a classification is, higher thresholds could be demanded. While this does not completely solve the issue (and may lead to an unacceptably low coverage rate), the risks can be mitigated. From a practical point of view, classifications with *“low certainty”* could be flagged for human verification.

Some of the lowest levels of performance were seen for the group “findings,” which is also the group with the lowest IRA. This might indicate that questions that are less clear and difficult to answer for humans are ambiguous for LLMs as well.

It should be noted that the approach itself could, in principle, be applied to many other documents and scenarios (Fig. 1b). However, new evaluation on other datasets would be required to verify that the system achieves high levels of performance. As we have seen, a performant LLM-based system for data extraction from free text of mammography reports can be realized with a budget for the hardware components of less than 8000 CHF. While we used an NVIDIA RTX 6000 (Ada) with 48GB on-board RAM, the Rombos, Phi-4, Li, and Lamarck models could have also been executed on a smaller (and cheaper) GPU with 32GB on-board RAM. Of course, this cost estimation only includes the hardware and no personnel costs.

Using cloud-based models (e.g., GPT models accessible via the OpenAI API [16]), instead of local LLMs, would provide easy access to powerful models. Utilizing such powerful models could achieve higher performance levels. In a previous study with cloud-based models, we used different state-of-the-art LLMs, that achieved accuracies of > 90% in classifying the titles and abstracts of oncological trials into various categories [33]. However, for application to clinical documents with sensitive patient data, on-premise systems are needed to keep the data within the local hospital network.

The classification script itself is easy to deploy, using only a few lines of code [35]. The source code for the project is publicly available under an open-source license and could be implemented for other scenarios of data extraction after adjustment of the relevant topics and categories.

We hope that this work serves as a guide for researchers and clinicians interested in setting up a local LLM-based data extraction system. Active and practical participation of clinicians in the implementation of AI into healthcare systems will be crucial to ensure that systems meet real-world requirements and ultimately lead to the best possible outcome for patients [71].

### CDEs for Synoptic Reporting and IT Integration

The performance of the framework depends on the individual question asked (or CDE defined). Having a clear data structure is fundamental for structuring medical information. It further helps to identify which groups and CDEs the system performs well in and which it does not. CDEs are increasingly relevant for collecting medical data, not only within scientific trials, but also in clinical practice [24]. Particularly for RWD in radiology, CDEs are highly valuable, as they provide clear semantics and structure for documentation [72]. They are increasingly being used for the integration of radiological AI systems [73]. Due to the increasing relevance of CDEs in radiology, the Radiological Society of North America (RSNA), in collaboration with the ACR, has started developing their own repository of radiological CDEs [74]. CDEs can build a shared framework for synoptic reporting, similar to the datasets of the International Collaboration on Cancer Reporting (ICCR) that are used for structured reporting in pathology [75] (In principle, the ICCR datasets are equivalent to the CDE concept defined by the NIH).

Furthermore, as CDEs are directly machine-readable, they can be used for the implementation of information technology systems (including generative AI) [76]. LLM-based as well as classical NLP systems for data extraction can directly be connected [77]. As reported, the CDE concept can be successfully applied to the collection of RWD via implementation into clinical IT and AI systems [78], [29].

CDEs provide an ideal means to support data interoperability. Rather than seeing radiology reports as a separate entity, information from different medical disciplines can be converted into CDE values. Combining extracted CDE values from various diagnostic and therapeutic paths thus may be a solution to address the challenges of data-driven medicine [25].

### Limitations

Our study has several limitations. In comparison to other approaches for data extraction, like rule-based systems or named-entity recognition (NER), LLMs are black boxes, and it is very challenging to understand exactly why a conclusion was reached by the model. There might be ways to address this using systems like the LLM transparency tool of meta [79], but the issue cannot be completely solved. Furthermore, LLMs are much more resource-intensive and some of the CDE values in our study could probably also have been accurately extracted using rule-based systems.

It should also be noted that the script used in this study can only extract categorical data values (“value list CDEs”). To retrieve other relevant information (e.g., numeric values), further adaptation would be needed.

Furthermore, while 61 mammography reports from clinical practice were used, no external validation of the performance with a second independent dataset was performed. While 1533 classification tasks were performed by two independent researchers and the LLMs, the number of classifications for each individual CDE is limited, which restricts the ability to draw more comprehensive conclusions. Generalization of the results may therefore be limited. We hypothesize that the general approach would work well in other settings, as the used LLMs are general models and already perform well without specific optimization for our use-case. Nevertheless, the results should be interpreted with caution, as it is unclear how well the approach would work on other data or in other scenarios. Creating ground truth datasets is labor-intensive and requires skilled personnel with medical knowledge. Since the patient-related data is sensitive, obtaining and sharing such valuable datasets for validation studies is often not possible. It should be noted that the LLMs used in the study were not specifically trained on internal data. The recent literature on LLM-based data extraction suggests that this kind of approach could successfully work in various settings [32].

One strength of our study is the clear definition of data concepts within a structured hierarchy using the CDE concept. If clear data standards are used on a broader level, this can be used to facilitate interoperability, clear semantics, and the comparison of results.

## Conclusions

Locally deployed, open-source LLMs can effectively extract information from unstructured mammography reports for structuring within a CDE-based framework. By retaining all data locally, the approach addresses key data privacy concerns and remains compatible with settings that have limited computational resources. Prompt engineering techniques are crucial, as they heavily influence the performance of the system. Going forward, wider adoption of standardized data definitions and locally implementable LLMs holds promise for enhancing interoperability, streamlining clinical workflows, and advancing research through reliable and secure data extraction.

## Supplementary Information

Below is the link to the electronic supplementary material.

Supplementary file1 (DOCX 488 KB)

Supplementary file2 (DOCX 85 KB)

Supplementary file3 (DOCX 26.2 KB)

## Data Availability

The source code for the project, as well as the .JSON files containing the relevant data about the data structure with classification topics, categories, and prompts, are provided at *GitHub* (35)*.* Information regarding the used anonymized mammography reports can be obtained from the authors upon reasonable request.

## Abbreviations

ACR: American college of radiology
AI: Artificial intelligence
API: Application programming interface
CDE: Common data element
CoT: Chain of thought
IRA: Inter-rater agreement
IT: Information technology
LLM: Large language model
LoRA: Low-rank adaptation
NER: Named entity recognition
NIH: National Institutes of Health
NLP: Natural language processing
RSNA: Radiological Society of North America
RWD: Real-world data
