# Supplementary material for: Implementing a Resource-Light and Low-Code Large Language Model System for Information Extraction from Mammography Reports: A Pilot Study
Source: J Imaging Inform Med. 2025 Sep 10;39(3):2737–51. doi: 10.1007/s10278-025-01659-4 (PMC13230402; doi:10.1007/s10278-025-01659-4)

**Supplementary Figure 1**: Schematic overview of the methodology used in the study. The CDE-based data structure was conceptualized by an interdisciplinary expert panel. The corresponding file for import into the general-classifier framework was provided in JSON. Two independent physicians manually assigned the values according to the structure for the mammography reports. Based on this manual data assignment, a ground truth dataset (in .CSV) was created after resolving cases of disagreement by the study coordination. Using the *general-classifier* python library, these two files were utilized to execute an LLM for data extraction on a local server. Evaluation runs with different LLMs and prompting techniques were done in the experiments. Statistical analyses with performance metrics and test for statistical significance using bootstrap resampling was conducted.

**
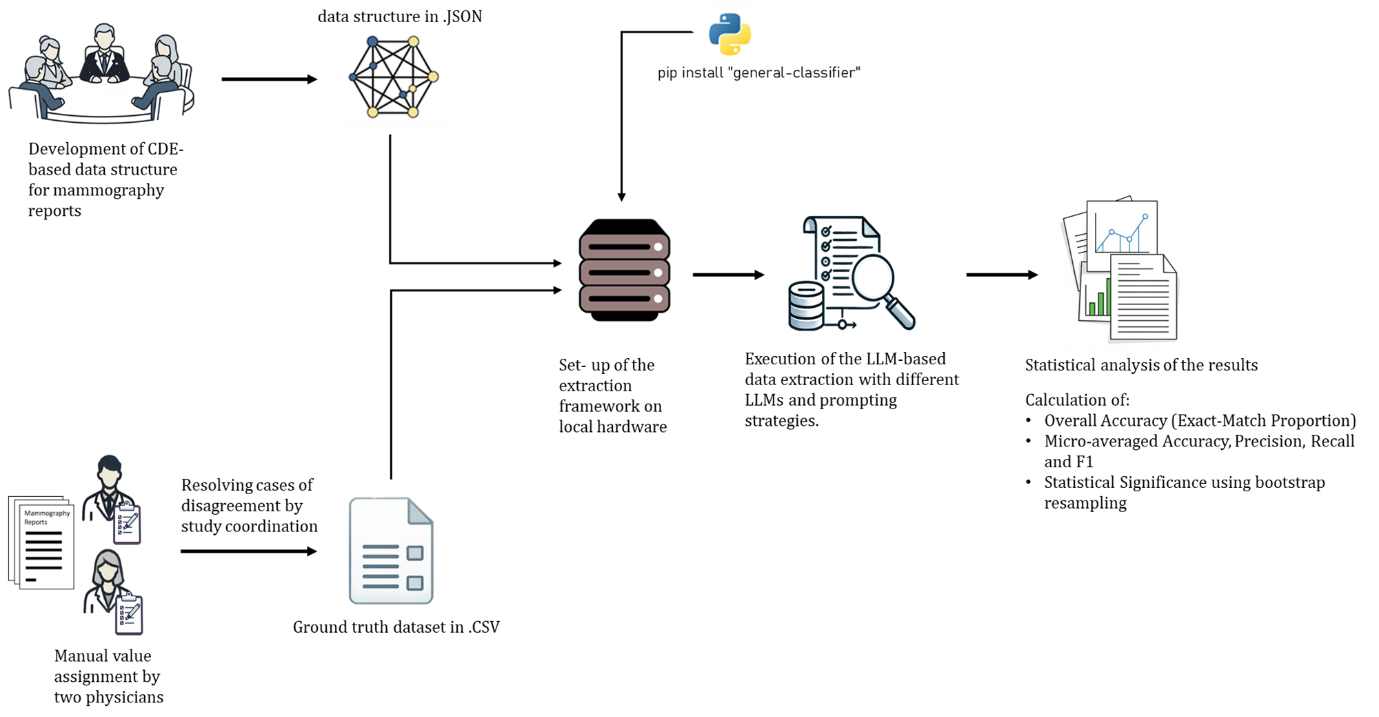
**

**Supplementary Figure 2**: Micro-averaged Recall of the LLM-based classification system with the five LLMs on the different groups, sub-groups and overall in classifying the mammography reports. Results for the default prompt.


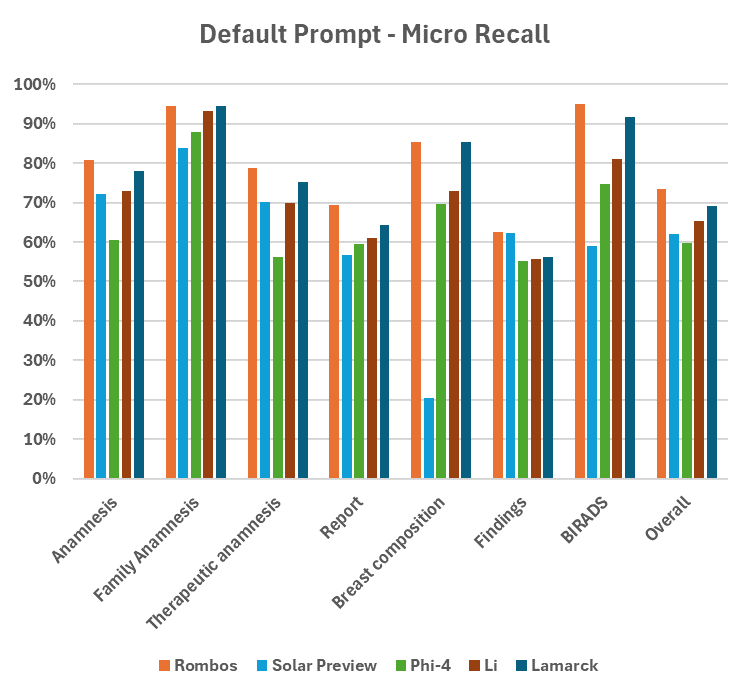


**Supplementary Figure 3**: Macro-averaged Recall of the LLM-based classification system with the five LLMs on the different groups, sub-groups and overall in classifying the mammography reports. Results for the default prompt.


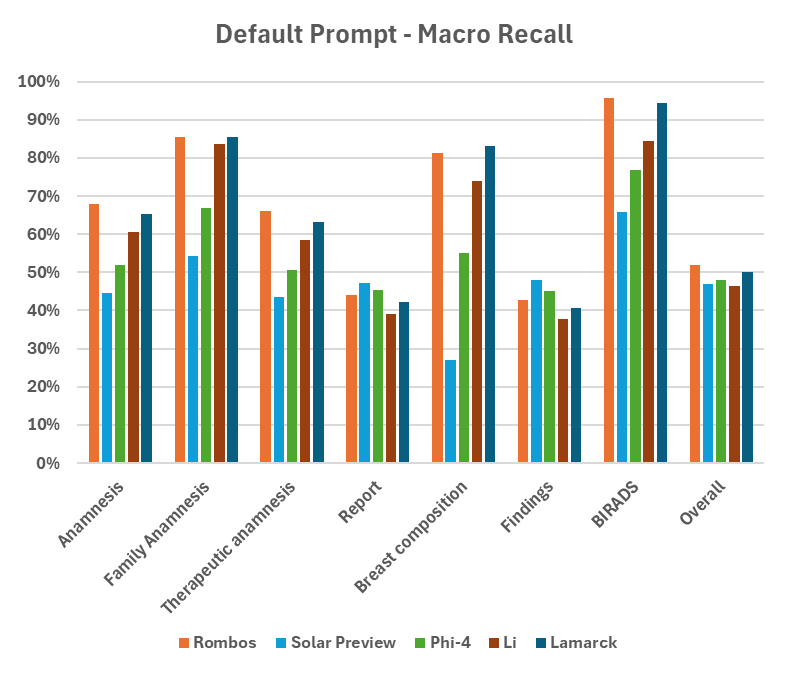


**Supplementary Figure 4**: Micro-averaged Precision of the LLM-based classification system with the five LLMs on the different groups, sub-groups and overall in classifying the mammography reports. Results for the default prompt.


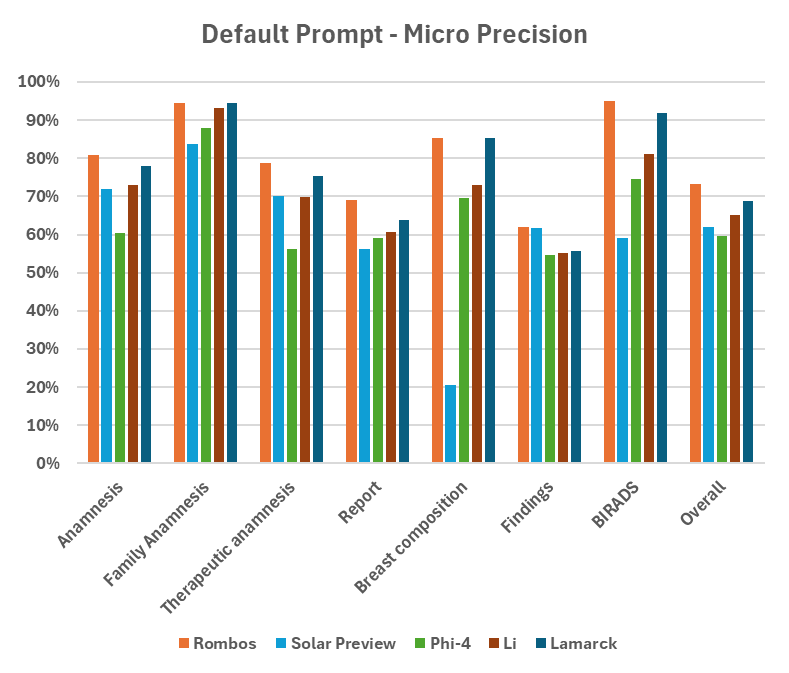


**Supplementary Figure 5**: Macro-averaged Precision of the LLM-based classification system with the five LLMs on the different groups, sub-groups and overall in classifying the mammography reports. Results for the default prompt.


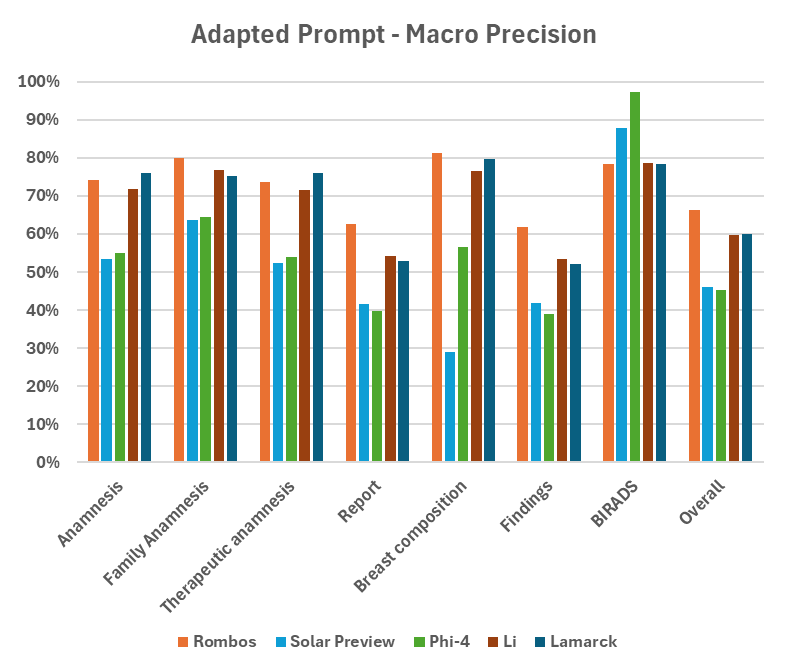


**Supplementary Figure 6**: Micro-averaged F1 of the LLM-based classification system with the five LLMs on the different groups, sub-groups and overall in classifying the mammography reports. Results for the default prompt.


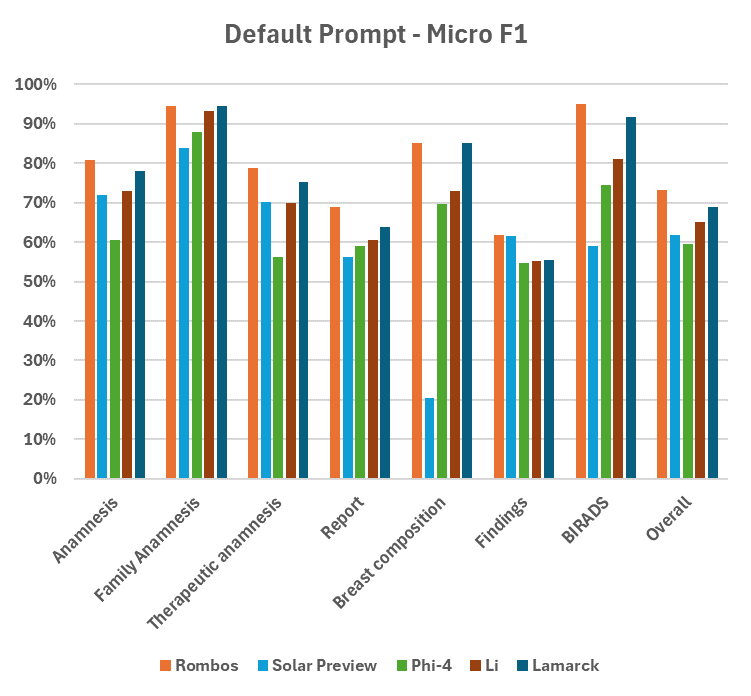


**Supplementary Figure 7**: Macro-averaged F1 of the LLM-based classification system with the five LLMs on the different groups, sub-groups and overall in classifying the mammography reports. Results for the default prompt.


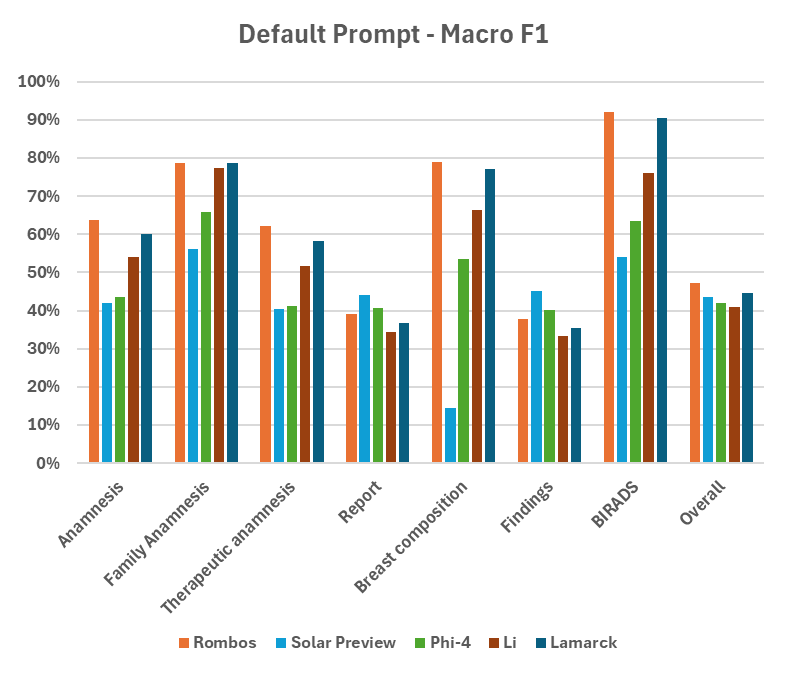


**Supplementary Figure 8**: Micro-averaged Recall of the LLM-based classification system with the five LLMs on the different groups, sub-groups and overall in classifying the mammography reports. Results for the adapted prompt.


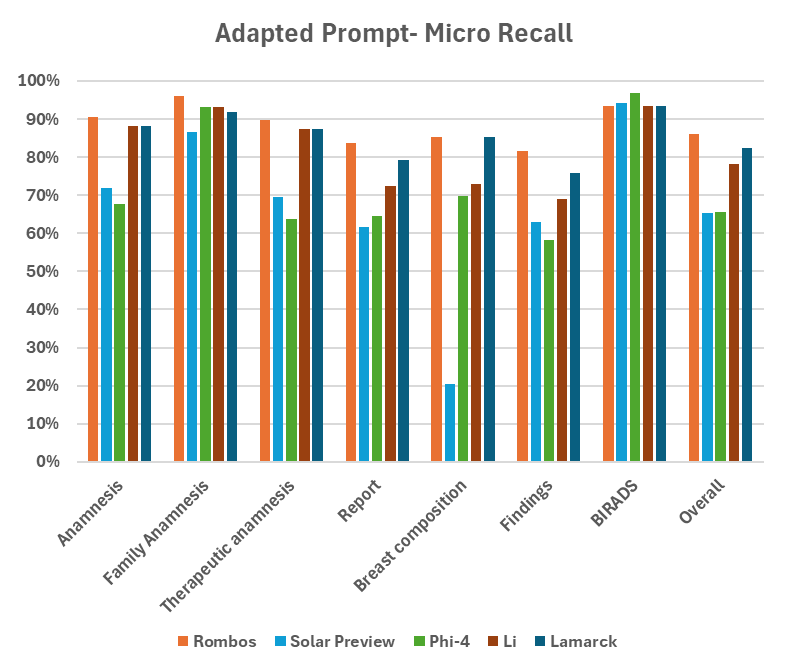


**Supplementary Figure 9**: Macro-averaged Recall of the LLM-based classification system with the five LLMs on the different groups, sub-groups and overall in classifying the mammography reports. Results for the adapted prompt.


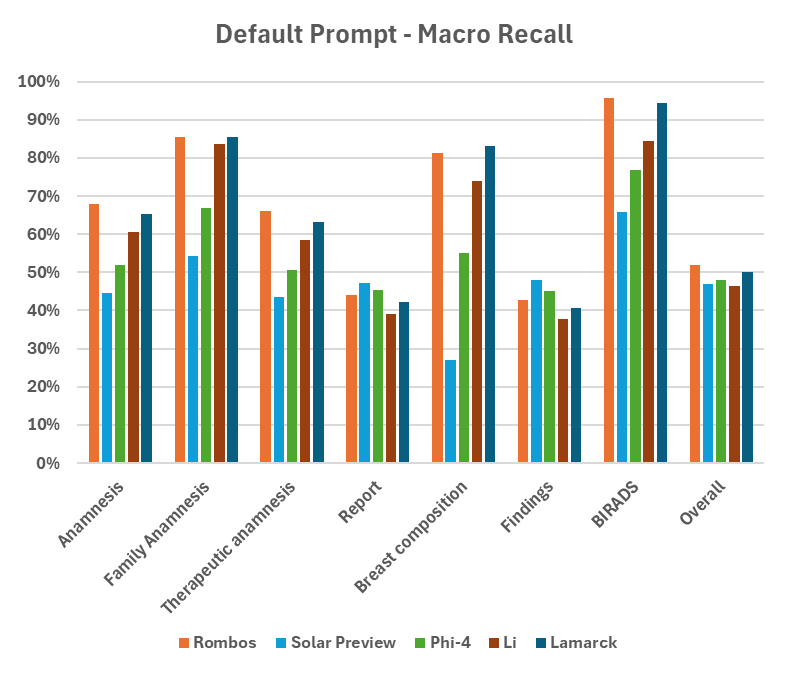


**Supplementary Figure 10**: Micro-averaged Precision of the LLM-based classification system with the five LLMs on the different groups, sub-groups and overall in classifying the mammography reports. Results for the adapted prompt.


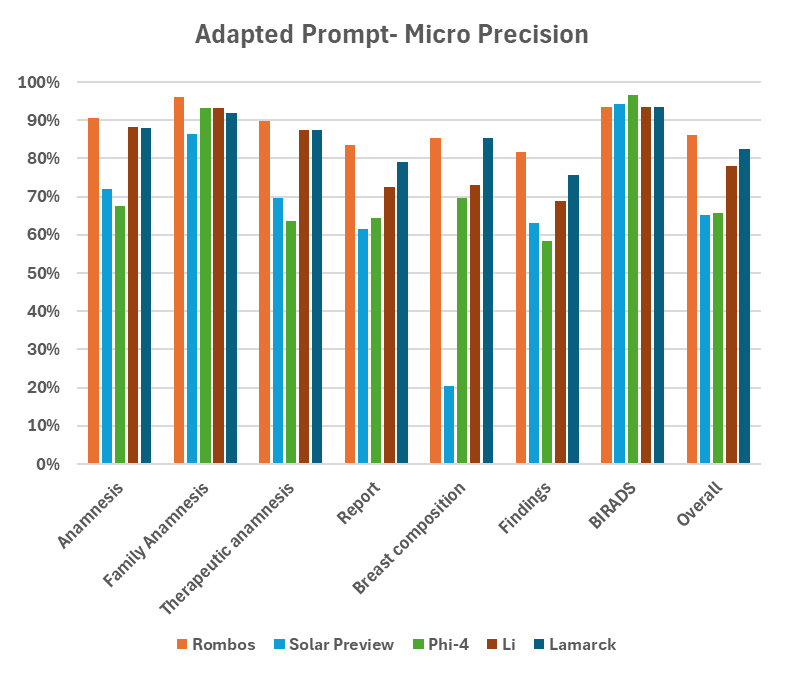


**Supplementary Figure 11**: Macro-averaged Precision of the LLM-based classification system with the five LLMs on the different groups, sub-groups and overall in classifying the mammography reports. Results for the adapted prompt.


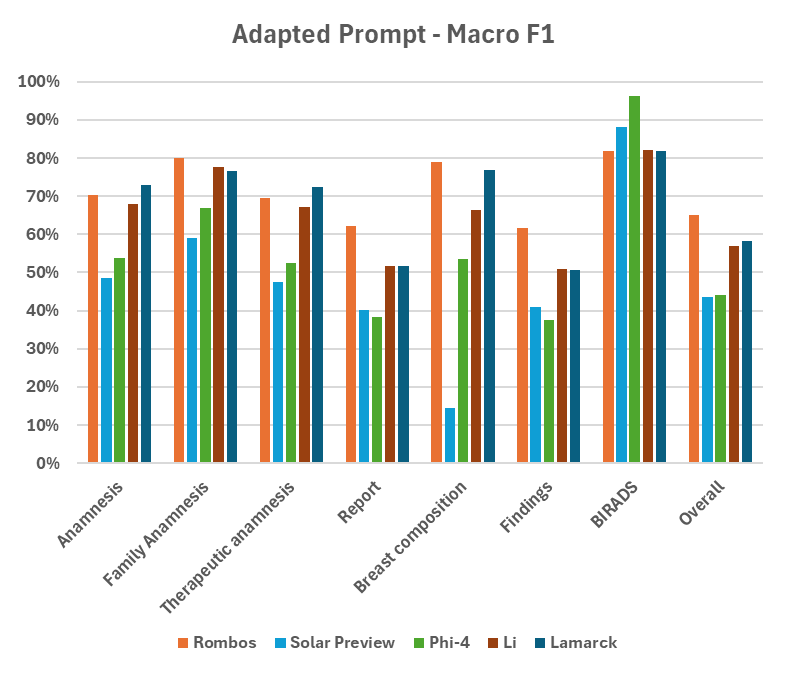


**Supplementary Figure 12**: Micro-averaged F1 of the LLM-based classification system with the five LLMs on the different groups, sub-groups and overall in classifying the mammography reports. Results for the adapted prompt.


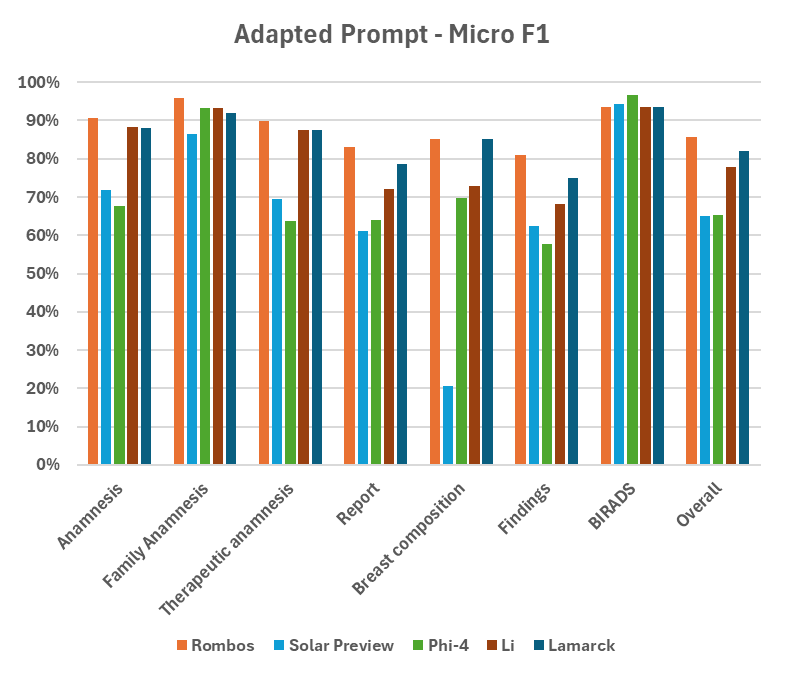


**Supplementary Figure 13**: Macro-averaged F1 of the LLM-based classification system with the five LLMs on the different groups, sub-groups and overall in classifying the mammography reports. Results for the adapted prompt.


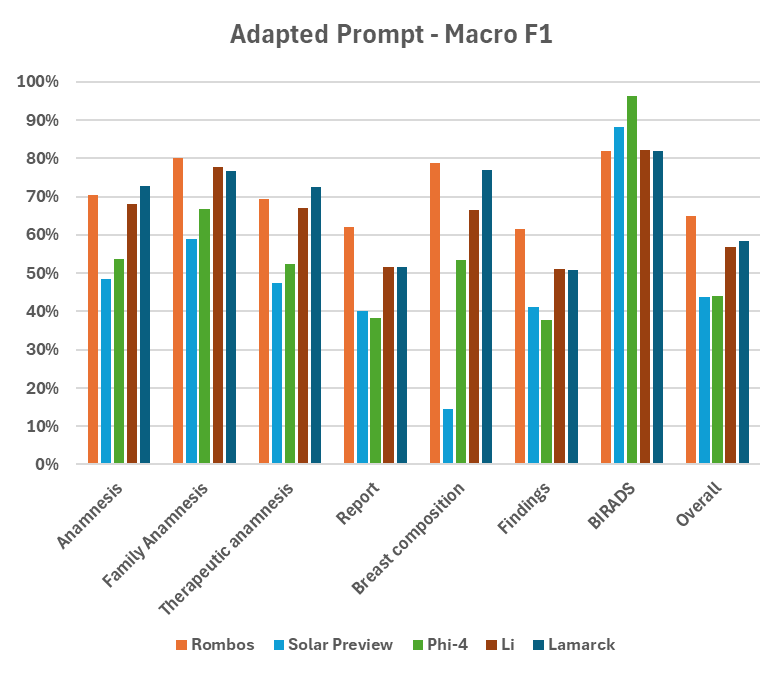


**Supplementary Figure 14**: Rate of accuracy for the classifications using the default prompt depending on the threshold for the relative probability


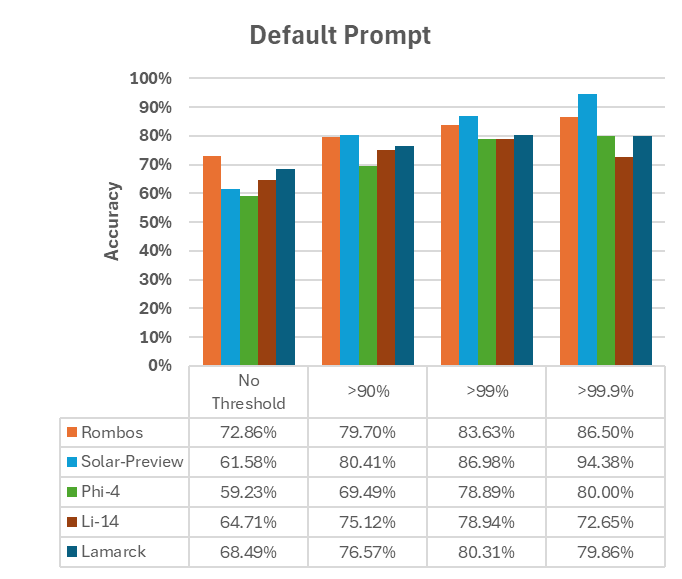


**Supplementary Figure 15**: Coverage rate for the classifications using the default prompt depending on the threshold for the relative probability


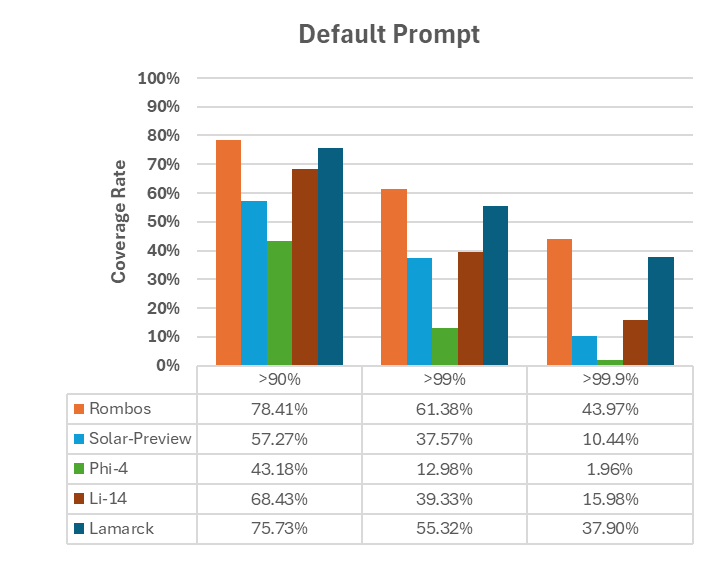


**Supplementary Figure 16**: Rate of accuracy for the classifications using the adapted prompt depending on the threshold for the relative probability


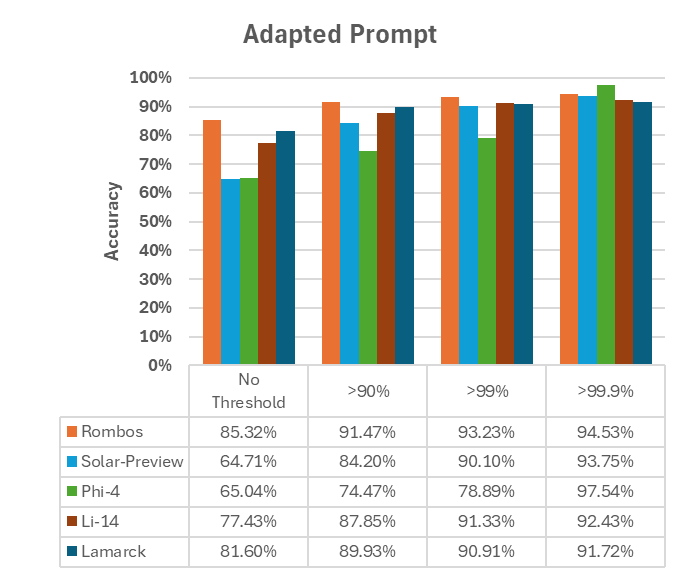


**Supplementary Figure 17**: Coverage for the classifications using the adapted prompt depending on the threshold for the relative probability


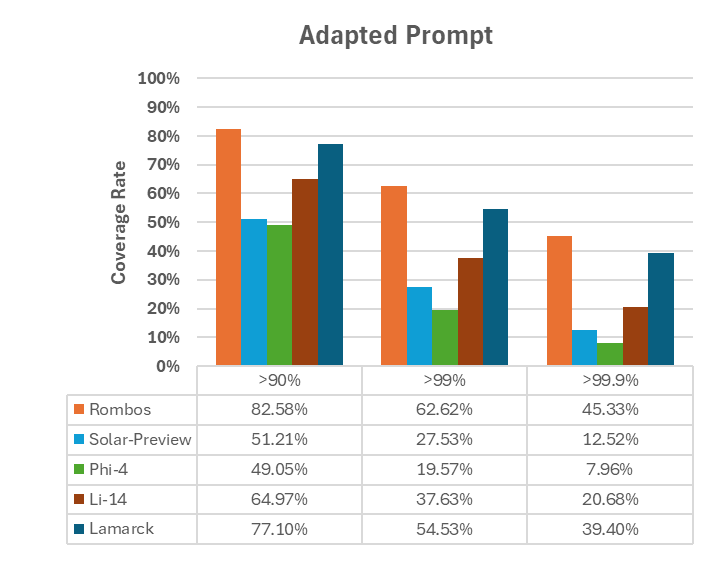

Supplement: Supplementary file 1 — Supplementary file1 (DOCX 488 KB) [file 10278_2025_1659_MOESM1_ESM.docx]
