# Supplementary material for: Implementing a Resource-Light and Low-Code Large Language Model System for Information Extraction from Mammography Reports: A Pilot Study
Source: J Imaging Inform Med. 2025 Sep 10;39(3):2737–51. doi: 10.1007/s10278-025-01659-4 (PMC13230402; doi:10.1007/s10278-025-01659-4)
Supplement: Supplementary file 2 — Supplementary file2 (DOCX 85 KB) [file 10278_2025_1659_MOESM2_ESM.docx]

Supplementary Table 1: Details on the datasets of the mammography reports.

|  | | **Absolute** | **Relative** |
| --- | --- | --- | --- |
| **Reports with at least one mentioned lesion** | | 25 | 41.0% |
| **Number of lesions in all reports** | | 34 | 55.7% |
| **Known breast cancer in medical history** | | 16 | 26.2 |
| **BI-RADS Left Breast** | |  |  |
|  | 0 | 5 | 8.3% |
|  | 1 | 24 | 40.0% |
|  | 2 | 15 | 25.0% |
|  | 3 | 5 | 8.3% |
|  | 4 | 5 | 8.3% |
|  | 5 | 4 | 6.7% |
|  | Not mentioned in the report | 2 | 3.3% |
| **BI-RADS Right Breast** | |  |  |
|  | 0 | 1 | 1.8% |
|  | 1 | 25 | 45.5% |
|  | 2 | 15 | 27.3% |
|  | 3 | 4 | 7.3% |
|  | 4 | 5 | 9.1% |
|  | 5 | 3 | 5.5% |
|  | Not mentioned in the report | 2 | 3.6% |
| **ACR Left Breast** | |  |  |
|  | A | 9 | 14.8% |
|  | B | 22 | 36.1% |
|  | C | 23 | 37.7% |
|  | D | 6 | 9.8% |
|  | Not mentioned in the report | 1 | 1.6% |
| **ACR Right Breast** | |  |  |
|  | A | 10 | 16.4% |
|  | B | 21 | 34.4% |
|  | C | 23 | 37.7% |
|  | D | 6 | 9.8% |
|  | Not mentioned in the report | 1 | 1.6% |

Supplementary Table 2: Inter-Rater Agreement between the two researchers assigning the CDE values calculating Cohen’s kappa. *For some CDEs Cohen’s kappa cannot be calculated due to low occurrence in the analyzed data set with not all values appearing in the data

|  | | | **Cohen’s Kappa** |
| --- | --- | --- | --- |
| **Anamnesis** | | | 0.90 |
|  | **Family anamnesis** | | 0.94 |
|  |  | Positive family anamnesis mentioned | 0.95 |
|  |  | Relative with breast cancer | 0.89 |
|  |  | |  |
|  | **Therapeutic anamnesis** | | 0.90 |
|  |  | Known breast cancer | 1.00 |
|  |  | Laterality of known breast cancer | 1.00 |
|  |  | Previous irradiation | 0.72 |
|  |  | Previous chemotherapy | 0.68 |
|  |  | Previous surgery | 0.88 |
|  |  | Second known breast cancer | 1.00 |
|  |  | Laterality of second known breast cancer | -* |
|  |  | Irradiation of second known breast cancer | 0.00 |
|  |  | Chemotherapy for second known breast cancer | -* |
|  |  | Surgery for second known breast cancer | -* |
|  |  | Non-oncological surgery conducted | 0.31 |
|  |  | Previous mammography conducted | 0.90 |
|  |  | Previous MR-mammography conducted | 0.73 |
|  |  | Previous biopsy conducted | 0.85 |
|  |  | Laterality of previously conducted biopsy | 1.00 |
|  |  | Quadrant of previously conducted biopsy | 0.00 |
|  |  | Clock position of previously conducted biopsy | 1.00 |
|  |  | Depth of previously conducted biopsy | -* |
|  |  | Result of previously conducted biopsy | 1.00 |
|  |  | Previous sonography conducted | 0.87 |
| **Report** | | |  |
|  | **Breast composition** | | 0.92 |
|  |  | ACR right breast | 0.91 |
|  |  | ACR left breast | 0.93 |
|  | **Findings** | | 0.71 |
|  |  | Lesion mentioned | 0.80 |
|  |  | Shape of the first lesion mentioned | 0.47 |
|  |  | Margin of the first lesion mentioned | 0.69 |
|  |  | Density of the first lesion mentioned | 0.43 |
|  |  | Dynamic of the first lesion mentioned | 0.76 |
|  |  | Mentioning of associated calcifications of the first lesion mentioned | 0.52 |
|  |  | Laterality of the first lesion mentioned | 0.92 |
|  |  | Visibility on view of the first lesion mentioned | 0.00 |
|  |  | Quadrant of the first lesion mentioned | 0.07 |
|  |  | Clock position of the first lesion mentioned | 0.72 |
|  |  | Depth of the first lesion mentioned | 0.80 |
|  |  | Second lesion mentioned | 0.90 |
|  |  | Shape of the second lesion mentioned | 0.60 |
|  |  | Margin of the second lesion mentioned | 0.59 |
|  |  | Density of the second lesion mentioned | 0.00 |
|  |  | Dynamic of the second lesion mentioned | 0.45 |
|  |  | Associated calcification mentioned of the second lesion mentioned | 0.75 |
|  |  | Laterality of the second lesion mentioned | 0.81 |
|  |  | Visibility on view of the second lesion mentioned | 0.00 |
|  |  | Quadrant of the second lesion mentioned | 0.37 |
|  |  | Clock position of the second lesion mentioned | 0.79 |
|  |  | Depth of the second lesion mentioned | 0.00 |
|  |  | Third lesion mentioned | 0.00 |
|  |  | Shape of the third lesion mentioned | -* |
|  |  | Margin of the third lesion mentioned | -* |
|  |  | Density of the third lesion mentioned | -* |
|  |  | Dynamic of the third lesion mentioned | 0.00 |
|  |  | Associated calcification of the third lesion mentioned | 0.00 |
|  |  | Laterality of the third lesion mentioned | 0.00 |
|  |  | Visibility on view of the third lesion mentioned | -* |
|  |  | Quadrant of the third lesion mentioned | 0.00 |
|  |  | Clock position of the third lesion mentioned | 0.00 |
|  |  | Depth of the third lesion mentioned | -* |
|  |  | Asymmetry mentioned | 0.91 |
|  |  | Description of mentioned asymmetry | 0.33 |
|  |  | Associated calcification of mentioned asymmetry | 1.00 |
|  |  | Laterality of mentioned asymmetry | 1.00 |
|  |  | Quadrant of mentioned asymmetry | 0.37 |
|  |  | Clock position of mentioned asymmetry | 1.00 |
|  |  | Depth of mentioned asymmetry | 0.00 |
|  |  | Architectural distorsion mentioned | 0.78 |
|  |  | Associated calcification of architectural distorsion mentioned | 0.67 |
|  |  | Depth of architectural distorsion | 0.00 |
|  |  | Laterality of architectural distorsion | 0.41 |
|  |  | Visibility on view of architectural distorsion | 0.00 |
|  |  | Quadrant of architectural distorsion | 0.00 |
|  |  | Clock position of architectural distorsion | 0.33 |
|  |  | Intramammary lymph node mentioned | 0.38 |
|  |  | Laterality of intramammary lymph node | 0.00 |
|  |  | Quadrant of intramammary lymph node | 0.00 |
|  |  | Clock position of intramammary lymph node | -* |
|  |  | Depth of intramammary lymph node | 0.00 |
|  |  | Solitary dilated duct | -* |
|  | **BIRADS** | | 0.95 |
|  |  | BIRADS left breast | 0.98 |
|  |  | BIRADS right breast | 0.92 |
|  | | | |
| **Overall** | | | 0.83 |

Supplementary Table 3: Overall Accuracies of the different LLMs on the individual CDEs

|  | | | **n** | **Rombos** | **Solar Preview** | **Phi-4** | **Li-14** | **Lamarck** |
| --- | --- | --- | --- | --- | --- | --- | --- | --- |
| **Anamnesis** | | | 544 | 80.9% | 72.1% | 60.5% | 73.0% | 77.9% |
|  | **Family anamnesis** | | 74 | 94.6% | 83.8% | 87.8% | 93.2% | 94.6% |
|  |  | Positive family anamnesis mentioned | 61 | 98.4% | 93.4% | 91.8% | 96.7% | 98.4% |
|  |  | Relative with breast cancer | 13 | 76.9% | 38.5% | 69.2% | 76.9% | 76.9% |
|  | **Therapeutic anamnesis** | | 470 | 78.7% | 70.2% | 56.2% | 69.8% | 75.3% |
|  |  | Known breast cancer | 61 | 82.0% | 83.6% | 57.4% | 65.6% | 78.7% |
|  |  | Laterality of known breast cancer | 16 | 68.8% | 56.3% | 56.3% | 62.5% | 68.8% |
|  |  | Previous irradiation | 16 | 68.8% | 75.0% | 25.0% | 62.5% | 68.8% |
|  |  | Previous chemotherapy | 16 | 75.0% | 75.0% | 56.3% | 62.5% | 68.8% |
|  |  | Previous surgery | 16 | 100.0% | 68.8% | 62.5% | 62.5% | 93.8% |
|  |  | Second known breast cancer | 16 | 43.8% | 93.8% | 31.3% | 37.5% | 31.3% |
|  |  | Laterality of second known breast cancer | 1 | 0.0% | 0.0% | 0.0% | 100.0% | 0.0% |
|  |  | Irradiation of second known breast cancer | 1 | 100.0% | 0.0% | 100.0% | 100.0% | 100.0% |
|  |  | Chemotherapy for second known breast cancer | 1 | 100.0% | 100.0% | 0.0% | 0.0% | 100.0% |
|  |  | Surgery for second known breast cancer | 1 | 100.0% | 0.0% | 100.0% | 100.0% | 100.0% |
|  |  | Non-oncological surgery conducted | 61 | 90.2% | 93.4% | 85.3% | 85.3% | 82.0% |
|  |  | Previous mammography conducted | 61 | 72.1% | 55.7% | 59.0% | 57.4% | 67.2% |
|  |  | Previous MR-mammography conducted | 61 | 85.3% | 67.2% | 34.4% | 85.3% | 83.6% |
|  |  | Previous biopsy conducted | 61 | 88.5% | 93.4% | 63.9% | 82.0% | 83.6% |
|  |  | Laterality of previously conducted biopsy | 4 | 25.0% | 25.0% | 50.0% | 50.0% | 25.0% |
|  |  | Quadrant of previously conducted biopsy | 4 | 25.0% | 0.0% | 25.0% | 25.0% | 25.0% |
|  |  | Clock position of previously conducted biopsy | 4 | 50.0% | 0.0% | 25.0% | 25.0% | 25.0% |
|  |  | Depth of previously conducted biopsy | 4 | 75.0% | 25.0% | 25.0% | 50.0% | 25.0% |
|  |  | Result of previously conducted biopsy | 4 | 50.0% | 25.0% | 50.0% | 25.0% | 75.0% |
|  |  | Previous sonography conducted | 61 | 75.4% | 44.3% | 57.4% | 70.5% | 82.0% |
| **Report** | | | 989 | 68.5% | 55.8% | 58.5% | 60.2% | 63.3% |
|  | **Breast composition** | | 122 | 85.3% | 20.5% | 69.7% | 73.0% | 85.3% |
|  |  | ACR right breast | 61 | 83.6% | 19.7% | 67.2% | 70.5% | 86.9% |
|  |  | ACR left breast | 61 | 86.9% | 21.3% | 72.1% | 75.4% | 83.6% |
|  | **Findings** | | 745 | 61.3 | 61.1% | 54.1% | 54.6% | 55.0% |
|  |  | Lesion mentioned | 61 | 78.7% | 65.6% | 50.8% | 72.1% | 70.5% |
|  |  | Shape of the first lesion mentioned | 25 | 72.0% | 64.0% | 64.0% | 52.0% | 56.0% |
|  |  | Margin of the first lesion mentioned | 25 | 64.0% | 20.0% | 44.0% | 56.0% | 64.0% |
|  |  | Density of the first lesion mentioned | 25 | 76.0% | 20.0% | 28.0% | 64.0% | 80.0% |
|  |  | Dynamic of the first lesion mentioned | 25 | 28.0% | 32.0% | 44.0% | 16.0% | 24.0% |
|  |  | Mentioning of associated calcifications of the first lesion mentioned | 25 | 24.0% | 60.0% | 28.0% | 28.0% | 24.0% |
|  |  | Laterality of the first lesion mentioned | 25 | 96.0% | 88.0% | 84.0% | 96.0% | 96.0% |
|  |  | Visibility on view of the first lesion mentioned | 25 | 56.0% | 36.0% | 88.0% | 44.0% | 52.0% |
|  |  | Quadrant of the first lesion mentioned | 25 | 48.0% | 36.0% | 48.0% | 28.0% | 44.0% |
|  |  | Clock position of the first lesion mentioned | 25 | 16.0% | 12.0% | 52.0% | 32.0% | 16.0% |
|  |  | Depth of the first lesion mentioned | 25 | 52.0% | 40.0% | 24.0% | 44.0% | 40.0% |
|  |  | Second lesion mentioned | 25 | 64.0% | 76.0% | 32.0% | 36.0% | 36.0% |
|  |  | Shape of the second lesion mentioned | 8 | 62.5% | 100.0% | 62.5% | 50.0% | 62.5% |
|  |  | Margin of the second lesion mentioned | 8 | 37.5% | 0.0% | 37.5% | 37.5% | 37.5% |
|  |  | Density of the second lesion mentioned | 8 | 75.0% | 62.5% | 75.0% | 87.5% | 75.0% |
|  |  | Dynamic of the second lesion mentioned | 8 | 12.5% | 25.0% | 37.5% | 12.5% | 12.5% |
|  |  | Associated calcification mentioned of the second lesion mentioned | 8 | 62.5% | 37.5% | 25.0% | 50.0% | 37.5% |
|  |  | Laterality of the second lesion mentioned | 8 | 50.0% | 50.0% | 37.5% | 37.5% | 50.0% |
|  |  | Visibility on view of the second lesion mentioned | 8 | 50.0% | 25.0% | 87.5% | 25.0% | 62.5% |
|  |  | Quadrant of the second lesion mentioned | 8 | 25.0% | 12.5% | 25.0% | 25.0% | 25.0% |
|  |  | Clock position of the second lesion mentioned | 8 | 12.5% | 0.0% | 62.5% | 12.5% | 0.0% |
|  |  | Depth of the second lesion mentioned | 8 | 37.5% | 25.0% | 37.5% | 25.0% | 25.0% |
|  |  | Third lesion mentioned | 1 | 100.0% | 100.0% | 100.0% | 100.0% | 100.0% |
|  |  | Shape of the third lesion mentioned | 1 | 100.0% | 100.0% | 100.0% | 100.0% | 100.0% |
|  |  | Margin of the third lesion mentioned | 1 | 100.0% | 0.0% | 0.0% | 100.0% | 100.0% |
|  |  | Density of the third lesion mentioned | 1 | 100.0% | 100.0% | 100.0% | 100.0% | 100.0% |
|  |  | Dynamic of the third lesion mentioned | 1 | 0.0% | 0.0% | 0.0% | 0.0% | 0.0% |
|  |  | Associated calcification of the third lesion mentioned | 1 | 0.0% | 100.0% | 0.0% | 0.0% | 0.0% |
|  |  | Laterality of the third lesion mentioned | 1 | 0.0% | 100.0% | 100.0% | 0.0% | 0.0% |
|  |  | Visibility on view of the third lesion mentioned | 1 | 100.0% | 100.0% | 100.0% | 100.0% | 100.0% |
|  |  | Quadrant of the third lesion mentioned | 1 | 0.0% | 0.0% | 0.0% | 0.0% | 0.0% |
|  |  | Clock position of the third lesion mentioned | 1 | 0.0% | 100.0% | 0.0% | 0.0% | 0.0% |
|  |  | Depth of the third lesion mentioned | 1 | 0.0% | 100.0% | 0.0% | 0.0% | 0.0% |
|  |  | Asymmetry mentioned | 61 | 47.5% | 85.3% | 32.8% | 27.9% | 27.9% |
|  |  | Description of mentioned asymmetry | 6 | 33.3% | 0.0% | 16.7% | 16.7% | 0.0% |
|  |  | Associated calcification of mentioned asymmetry | 6 | 33.3% | 50.0% | 50.0% | 33.3% | 33.3% |
|  |  | Laterality of mentioned asymmetry | 6 | 83.3% | 83.3% | 83.3% | 83.3% | 83.3% |
|  |  | Quadrant of mentioned asymmetry | 6 | 33.3% | 50.0% | 33.3% | 33.3% | 33.3% |
|  |  | Clock position of mentioned asymmetry | 6 | 16.7% | 16.7% | 50.0% | 33.3% | 16.7% |
|  |  | Depth of mentioned asymmetry | 6 | 16.7% | 16.7% | 16.7% | 16.7% | 16.7% |
|  |  | Architectural distorsion mentioned | 61 | 93.4% | 95.1% | 63.9% | 95.1% | 85.3% |
|  |  | Associated calcification of architectural distorsion mentioned | 5 | 0.0% | 100.0% | 40.0% | 0.0% | 0.0% |
|  |  | Depth of architectural distorsion | 5 | 60.0% | 40.0% | 40.0% | 40.0% | 40.0% |
|  |  | Laterality of architectural distorsion | 5 | 80.0% | 60.0% | 80.0% | 80.0% | 80.0% |
|  |  | Visibility on view of architectural distorsion | 5 | 20.0% | 20.0% | 80.0% | 40.0% | 20.0% |
|  |  | Quadrant of architectural distorsion | 5 | 20.0% | 20.0% | 20.0% | 0.0% | 20.0% |
|  |  | Clock position of architectural distorsion | 5 | 60.0% | 60.0% | 60.0% | 60.0% | 40.0% |
|  |  | Intramammary lymph node mentioned | 61 | 77.1% | 93.4% | 62.3% | 72.1% | 72.1% |
|  |  | Laterality of intramammary lymph node | 2 | 50.0% | 50.0% | 50.0% | 50.0% | 50.0% |
|  |  | Quadrant of intramammary lymph node | 2 | 0.0% | 50.0% | 50.0% | 0.0% | 0.0% |
|  |  | Clock position of intramammary lymph node | 2 | 0.0% | 0.0% | 50.0% | 0.0% | 50.0% |
|  |  | Depth of intramammary lymph node | 2 | 100.0% | 50.0% | 100.0% | 50.0% | 100.0% |
|  |  | Solitary dilated duct | 61 | 98.4% | 100.0% | 100.0% | 98.4% | 98.4% |
|  | **BIRADS** | | 122 | 95.1% | 59.0% | 74.6% | 81.2% | 91.8% |
|  |  | BIRADS left breast | 61 | 95.1% | 63.9% | 70.5% | 78.7% | 90.2% |
|  |  | BIRADS right breast | 61 | 95.1% | 54.1% | 78.7% | 83.6% | 93.4% |
| **Overall** | | | 1533 | 72.9% | 61.6% | 59.2% | 64.7% | 68.5% |

Supplementary Table 4: Statistical Analysis (Paired Bootstrap Results) for the Overall Accuracy. *statistically significant difference at the p < 0.005 accounting for the multiple comparisons problem with (10 pairs per metric).

|  | **Observed Difference** | **p-value** | **99.5% CI** |
| --- | --- | --- | --- |
| **Standard Prompt** | | | |
| Rombos vs. Solar-Preview | 0.1129 | <0.0001* | [0.0777, 0.1491] |
| Rombos vs. Phi-4 | 0.1363 | <0.0001* | [0.0950, 0.1740] |
| Rombos vs. Li-14 | 0.0815 | <0.0001* | [0.0539, 0.1059] |
| Rombos vs. Lamarck | 0.0437 | <0.0001* | [0.0247, 0.0634] |
| Solar-Preview vs. Phi-4 | 0.0235 | 0.1060 | [-0.0171, 0.0651] |
| Solar-Preview vs. Li-14 | -0.0313 | 0.0280 | [-0.0741, 0.0092] |
| Solar-Preview vs. Lamarck | -0.0691 | <0.0001* | [-0.1057, -0.0333] |
| Phi-4 vs. Li-14 | -0.0548 | <0.0001* | [-0.0955, -0.0175] |
| Phi-4 vs. Lamarck | -0.0926 | <0.0001* | [-0.1278, -0.0577] |
| Li-14 vs. Lamarck | -0.0378 | <0.0001* | [-0.0612, -0.0108] |
| **1-shot Prompt** | | | |
| Rombos vs. Solar-Preview | 0.2224 | <0.0001* | [0.1665, 0.2765] |
| Rombos vs. Phi-4 | 0.3666 | <0.0001* | [0.3201, 0.4199] |
| Rombos vs. Li-14 | 0.0313 | 0.0020* | [0.0013, 0.0636] |
| Rombos vs. Lamarck | 0.0254 | 0.0015* | [0.0028, 0.0490] |
| Solar-Preview vs. Phi-4 | 0.1442 | <0.0001* | [0.0890, 0.2030] |
| Solar-Preview vs. Li-14 | -0.1911 | <0.0001* | [-0.2497, -0.1392] |
| Solar-Preview vs. Lamarck | -0.1970 | <0.0001* | [-0.2486, -0.1470] |
| Phi-4 vs. Li-14 | -0.3353 | <0.0001* | [-0.3903, -0.2905] |
| Phi-4 vs. Lamarck | -0.3412 | <0.0001* | [-0.3885, -0.2966] |
| Li-14 vs. Lamarck | -0.0059 | 0.2720 | [-0.0322, 0.0199] |
| **3-shot Prompt** | | | |
| Rombos vs. Solar-Preview | -0.0137 | 0.1935 | [-0.0549, 0.0268] |
| Rombos vs. Phi-4 | 0.2485 | <0.0001* | [0.2059, 0.2950] |
| Rombos vs. Li-14 | 0.1194 | <0.0001* | [0.0702, 0.1701] |
| Rombos vs. Lamarck | 0.0176 | 0.0280 | [-0.0059, 0.0431] |
| Solar-Preview vs. Phi-4 | 0.2622 | <0.0001* | [0.2139, 0.3211] |
| Solar-Preview vs. Li-14 | 0.1331 | <0.0001* | [0.0713, 0.2077] |
| Solar-Preview vs. Lamarck | 0.0313 | 0.0230 | [-0.0120, 0.0749] |
| Phi-4 vs. Li-14 | -0.1292 | <0.0001* | [-0.1870, -0.0638] |
| Phi-4 vs. Lamarck | -0.2309 | <0.0001* | [-0.2805, -0.1819] |
| Li-14 vs. Lamarck | -0.1018 | <0.0001* | [-0.1605, -0.0513] |
| **CoT Prompt** | | | |
| Rombos vs. Solar-Preview | 0.2094 | <0.0001* | [0.1718, 0.2466] |
| Rombos vs. Phi-4 | 0.2648 | <0.0001* | [0.2121, 0.3187] |
| Rombos vs. Li-14 | 0.1005 | <0.0001* | [0.0675, 0.1408] |
| Rombos vs. Lamarck | 0.0385 | <0.0001* | [0.0170, 0.0606] |
| Solar-Preview vs. Phi-4 | 0.0554 | 0.0120 | [-0.0097, 0.1241] |
| Solar-Preview vs. Li-14 | -0.1089 | <0.0001* | [-0.1531, -0.0622] |
| Solar-Preview vs. Lamarck | -0.1709 | <0.0001* | [-0.2155, -0.1335] |
| Phi-4 vs. Li-14 | -0.1644 | <0.0001* | [-0.2201, -0.1086] |
| Phi-4 vs. Lamarck | -0.2264 | <0.0001* | [-0.2883, -0.1683] |
| Li-14 vs. Lamarck | -0.0620 | <0.0001* | [-0.1028, -0.0299] |
| **Adapted Prompt** | | | |
| Rombos vs. Solar-Preview | 0.2061 | <0.0001* | [0.1728, 0.2348] |
| Rombos vs. Phi-4 | 0.2029 | <0.0001* | [0.1704, 0.2360] |
| Rombos vs. Li-14 | 0.0789 | <0.0001* | [0.0500, 0.1065] |
| Rombos vs. Lamarck | 0.0372 | <0.0001* | [0.0163, 0.0608] |
| Solar-Preview vs. Phi-4 | -0.0033 | 0.4195 | [-0.0384, 0.0343] |
| Solar-Preview vs. Li-14 | -0.1272 | <0.0001* | [-0.1593, -0.0968] |
| Solar-Preview vs. Lamarck | -0.1689 | <0.0001* | [-0.1987, -0.1395] |
| Phi-4 vs. Li-14 | -0.1239 | <0.0001* | [-0.1552, -0.0913] |
| Phi-4 vs. Lamarck | -0.1657 | <0.0001* | [-0.2006, -0.1308] |
| Li-14 vs. Lamarck | -0.0417 | <0.0001* | [-0.0680, -0.0160] |

Supplementary Table 5: Statistical Analysis (Paired Bootstrap Results) for the Micro-averaged Recall. *statistically significant difference at the p < 0.005 accounting for the multiple comparisons problem with (10 pairs per metric).

|  | **Observed Difference** | **p-value** | **99.5% CI** |
| --- | --- | --- | --- |
| **Standard Prompt** | | | |
| Rombos vs. Solar-Preview | 0.1129 | <0.0001* | [0.0777, 0.1491] |
| Rombos vs. Phi-4 | 0.1363 | <0.0001* | [0.0950, 0.1740] |
| Rombos vs. Li-14 | 0.0815 | <0.0001* | [0.0539, 0.1059] |
| Rombos vs. Lamarck | 0.0437 | <0.0001* | [0.0247, 0.0634] |
| Solar-Preview vs. Phi-4 | 0.0235 | 0.1060 | [-0.0171, 0.0651] |
| Solar-Preview vs. Li-14 | -0.0313 | 0.0280 | [-0.0741, 0.0092] |
| Solar-Preview vs. Lamarck | -0.0691 | <0.0001* | [-0.1057, -0.0333] |
| Phi-4 vs. Li-14 | -0.0548 | <0.0001* | [-0.0955, -0.0175] |
| Phi-4 vs. Lamarck | -0.0926 | <0.0001* | [-0.1278, -0.0577] |
| Li-14 vs. Lamarck | -0.0378 | <0.0001* | [-0.0612, -0.0108] |
| **1-shot Prompt** | | | |
| Rombos vs. Solar-Preview | 0.2224 | <0.0001* | [0.1665, 0.2765] |
| Rombos vs. Phi-4 | 0.3666 | <0.0001* | [0.3201, 0.4199] |
| Rombos vs. Li-14 | 0.0313 | 0.0020* | [0.0013, 0.0636] |
| Rombos vs. Lamarck | 0.0254 | 0.0015* | [0.0028, 0.0490] |
| Solar-Preview vs. Phi-4 | 0.1442 | <0.0001* | [0.0890, 0.2030] |
| Solar-Preview vs. Li-14 | -0.1911 | <0.0001* | [-0.2497, -0.1392] |
| Solar-Preview vs. Lamarck | -0.1970 | <0.0001* | [-0.2486, -0.1470] |
| Phi-4 vs. Li-14 | -0.3353 | <0.0001* | [-0.3903, -0.2905] |
| Phi-4 vs. Lamarck | -0.3412 | <0.0001* | [-0.3885, -0.2966] |
| Li-14 vs. Lamarck | -0.0059 | 0.2720 | [-0.0322, 0.0199] |
| **3-shot Prompt** | | | |
| Rombos vs. Solar-Preview | -0.0137 | 0.1935 | [-0.0549, 0.0268] |
| Rombos vs. Phi-4 | 0.2485 | <0.0001* | [0.2059, 0.2950] |
| Rombos vs. Li-14 | 0.1194 | <0.0001* | [0.0702, 0.1701] |
| Rombos vs. Lamarck | 0.0176 | 0.0280 | [-0.0059, 0.0431] |
| Solar-Preview vs. Phi-4 | 0.2622 | <0.0001* | [0.2139, 0.3211] |
| Solar-Preview vs. Li-14 | 0.1331 | <0.0001* | [0.0713, 0.2077] |
| Solar-Preview vs. Lamarck | 0.0313 | 0.0230 | [-0.0120, 0.0749] |
| Phi-4 vs. Li-14 | -0.1292 | <0.0001* | [-0.1870, -0.0638] |
| Phi-4 vs. Lamarck | -0.2309 | <0.0001* | [-0.2805, -0.1819] |
| Li-14 vs. Lamarck | -0.1018 | <0.0001* | [-0.1605, -0.0513] |
| **CoT Prompt** | | | |
| Rombos vs. Solar-Preview | 0.2094 | <0.0001* | [0.1718, 0.2466] |
| Rombos vs. Phi-4 | 0.2648 | <0.0001* | [0.2121, 0.3187] |
| Rombos vs. Li-14 | 0.1005 | <0.0001* | [0.0675, 0.1408] |
| Rombos vs. Lamarck | 0.0385 | <0.0001* | [0.0170, 0.0606] |
| Solar-Preview vs. Phi-4 | 0.0554 | 0.0120 | [-0.0097, 0.1241] |
| Solar-Preview vs. Li-14 | -0.1089 | <0.0001* | [-0.1531, -0.0622] |
| Solar-Preview vs. Lamarck | -0.1709 | <0.0001* | [-0.2155, -0.1335] |
| Phi-4 vs. Li-14 | -0.1644 | <0.0001* | [-0.2201, -0.1086] |
| Phi-4 vs. Lamarck | -0.2264 | <0.0001* | [-0.2883, -0.1683] |
| Li-14 vs. Lamarck | -0.0620 | <0.0001* | [-0.1028, -0.0299] |
| **Adapted Prompt** | | | |
| Rombos vs. Solar-Preview | 0.2061 | <0.0001* | [0.1728, 0.2348] |
| Rombos vs. Phi-4 | 0.2029 | <0.0001* | [0.1704, 0.2360] |
| Rombos vs. Li-14 | 0.0789 | <0.0001* | [0.0500, 0.1065] |
| Rombos vs. Lamarck | 0.0372 | <0.0001* | [0.0163, 0.0608] |
| Solar-Preview vs. Phi-4 | -0.0033 | 0.4195 | [-0.0384, 0.0343] |
| Solar-Preview vs. Li-14 | -0.1272 | <0.0001* | [-0.1593, -0.0968] |
| Solar-Preview vs. Lamarck | -0.1689 | <0.0001* | [-0.1987, -0.1395] |
| Phi-4 vs. Li-14 | -0.1239 | <0.0001* | [-0.1552, -0.0913] |
| Phi-4 vs. Lamarck | -0.1657 | <0.0001* | [-0.2006, -0.1308] |
| Li-14 vs. Lamarck | -0.0417 | <0.0001* | [-0.0680, -0.0160] |

Supplementary Table 6: Statistical Analysis (Paired Bootstrap Results) for the Macro-averaged Recall. *statistically significant difference at the p < 0.005 accounting for the multiple comparisons problem with (10 pairs per metric).

|  | **Observed Difference** | **p-value** | **99.5% CI** |
| --- | --- | --- | --- |
| **Standard Prompt** | | | |
| Rombos vs. Solar-Preview | 0.0508 | 0.0320 | [-0.0183, 0.1508] |
| Rombos vs. Phi-4 | 0.0396 | 0.1180 | [-0.0275, 0.1019] |
| Rombos vs. Li-14 | 0.0576 | 0.0010* | [0.0151, 0.1016] |
| Rombos vs. Lamarck | 0.0203 | 0.0650 | [-0.0102, 0.0651] |
| Solar-Preview vs. Phi-4 | -0.0112 | 0.3030 | [-0.1168, 0.0383] |
| Solar-Preview vs. Li-14 | 0.0067 | 1.0170 | [-0.0993, 0.0785] |
| Solar-Preview vs. Lamarck | -0.0305 | 0.1840 | [-0.1199, 0.0373] |
| Phi-4 vs. Li-14 | 0.0179 | 0.3290 | [-0.0332, 0.0890] |
| Phi-4 vs. Lamarck | -0.0193 | 0.5260 | [-0.0600, 0.0409] |
| Li-14 vs. Lamarck | -0.0372 | 0.0500 | [-0.0767, 0.0200] |
| **1-shot Prompt** | | | |
| Rombos vs. Solar-Preview | 0.0508 | 0.032 | [-0.0183, 0.1508] |
| Rombos vs. Phi-4 | 0.0396 | 0.118 | [-0.0275, 0.1019] |
| Rombos vs. Li-14 | 0.0576 | 0.001 | [0.0151, 0.1016] |
| Rombos vs. Lamarck | 0.0203 | 0.065 | [-0.0102, 0.0651] |
| Solar-Preview vs. Phi-4 | -0.0112 | 0.303 | [-0.1168, 0.0383] |
| Solar-Preview vs. Li-14 | 0.0067 | 1.017 | [-0.0993, 0.0785] |
| Solar-Preview vs. Lamarck | -0.0305 | 0.184 | [-0.1199, 0.0373] |
| Phi-4 vs. Li-14 | 0.0179 | 0.329 | [-0.0332, 0.0890] |
| Phi-4 vs. Lamarck | -0.0193 | 0.526 | [-0.0600, 0.0409] |
| Li-14 vs. Lamarck | -0.0372 | 0.05 | [-0.0767, 0.0200] |
| **3-shot Prompt** | | | |
| Rombos vs. Solar-Preview | 0.1599 | <0.0001* | [0.0388, 0.1908] |
| Rombos vs. Phi-4 | 0.1742 | <0.0001* | [0.1194, 0.2255] |
| Rombos vs. Li-14 | 0.0164 | 0.068 | [-0.0235, 0.0739] |
| Rombos vs. Lamarck | 0.0061 | 0.1625 | [-0.0168, 0.0562] |
| Solar-Preview vs. Phi-4 | 0.0143 | 0.006 | [-0.0042, 0.1174] |
| Solar-Preview vs. Li-14 | -0.1435 | 0.002* | [-0.1684, -0.0103] |
| Solar-Preview vs. Lamarck | -0.1538 | <0.0001* | [-0.1791, -0.0269] |
| Phi-4 vs. Li-14 | -0.1579 | <0.0001* | [-0.2083, -0.0827] |
| Phi-4 vs. Lamarck | -0.1681 | <0.0001* | [-0.2132, -0.0902] |
| Li-14 vs. Lamarck | -0.0102 | 0.1805 | [-0.0523, 0.0214] |
| **CoT Prompt** | | | |
| Rombos vs. Solar-Preview | 0.1729 | <0.0001* | [0.1409, 0.2799] |
| Rombos vs. Phi-4 | 0.0957 | <0.0001* | [0.0500, 0.1969] |
| Rombos vs. Li-14 | 0.0941 | <0.0001* | [0.0383, 0.1429] |
| Rombos vs. Lamarck | 0.0801 | 0.0005* | [0.0079, 0.1194] |
| Solar-Preview vs. Phi-4 | -0.0773 | 0.0025* | [-0.1591, -0.0051] |
| Solar-Preview vs. Li-14 | -0.0788 | <0.0001* | [-0.1613, -0.0499] |
| Solar-Preview vs. Lamarck | -0.0928 | <0.0001* | [-0.2309, -0.0602] |
| Phi-4 vs. Li-14 | -0.0016 | 0.24 | [-0.1033, 0.0357] |
| Phi-4 vs. Lamarck | -0.0156 | 0.105 | [-0.1580, 0.0325] |
| Li-14 vs. Lamarck | -0.014 | 0.1495 | [-0.0860, 0.0268] |
| **Adapted Prompt** | | | |
| Rombos vs. Solar-Preview | 0.2078 | <0.0001* | [0.1577, 0.2588] |
| Rombos vs. Phi-4 | 0.1852 | <0.0001* | [0.1057, 0.2366] |
| Rombos vs. Li-14 | 0.0699 | <0.0001* | [0.0414, 0.1353] |
| Rombos vs. Lamarck | 0.0592 | 0.0095 | [-0.0082, 0.1071] |
| Solar-Preview vs. Phi-4 | -0.0226 | 0.069 | [-0.0917, 0.0202] |
| Solar-Preview vs. Li-14 | -0.1379 | <0.0001* | [-0.1817, -0.0643] |
| Solar-Preview vs. Lamarck | -0.1486 | <0.0001* | [-0.2246, -0.0998] |
| Phi-4 vs. Li-14 | -0.1153 | <0.0001* | [-0.1457, -0.0291] |
| Phi-4 vs. Lamarck | -0.126 | <0.0001* | [-0.1688, -0.0817] |
| Li-14 vs. Lamarck | -0.0107 | 0.0305 | [-0.0919, 0.0151] |

Supplementary Table 7: Statistical Analysis (Paired Bootstrap Results) for the Macro-averaged Precision. *statistically significant difference at the p < 0.005 accounting for the multiple comparisons problem with (10 pairs per metric).

|  | **Observed Difference** | **p-value** | **99.5% CI** |
| --- | --- | --- | --- |
| **Standard Prompt** | | | |
| Rombos vs. Solar-Preview | 0.0194 | 0.2000 | [-0.0366, 0.1369] |
| Rombos vs. Phi-4 | 0.0239 | 0.2880 | [-0.0374, 0.0956] |
| Rombos vs. Li-14 | 0.0394 | 0.0040* | [0.0014, 0.1011] |
| Rombos vs. Lamarck | 0.0155 | 0.0540 | [-0.0079, 0.0596] |
| Solar-Preview vs. Phi-4 | 0.0045 | 1.3230 | [-0.1048, 0.0468] |
| Solar-Preview vs. Li-14 | 0.0200 | 0.7800 | [-0.0876, 0.0944] |
| Solar-Preview vs. Lamarck | -0.0039 | 0.6000 | [-0.1029, 0.0524] |
| Phi-4 vs. Li-14 | 0.0155 | 0.3050 | [-0.0329, 0.0864] |
| Phi-4 vs. Lamarck | -0.0084 | 0.8800 | [-0.0513, 0.0531] |
| Li-14 vs. Lamarck | -0.0239 | 0.1430 | [-0.0773, 0.0304] |
| **1-shot Prompt** | | | |
| Rombos vs. Solar-Preview | 0.1343 | 0.0005* | [0.0305, 0.2321] |
| Rombos vs. Phi-4 | 0.1753 | <0.0001* | [0.1298, 0.2608] |
| Rombos vs. Li-14 | 0.0125 | 0.312 | [-0.0487, 0.0605] |
| Rombos vs. Lamarck | -0.0514 | 0.0425 | [-0.0713, 0.0231] |
| Solar-Preview vs. Phi-4 | 0.041 | 0.019 | [-0.0274, 0.1439] |
| Solar-Preview vs. Li-14 | -0.1218 | <0.0001* | [-0.1940, -0.0385] |
| Solar-Preview vs. Lamarck | -0.1857 | <0.0001* | [-0.2404, -0.0618] |
| Phi-4 vs. Li-14 | -0.1629 | <0.0001* | [-0.2448, -0.1210] |
| Phi-4 vs. Lamarck | -0.2268 | <0.0001* | [-0.2834, -0.1501] |
| Li-14 vs. Lamarck | -0.0639 | 0.08 | [-0.0884, 0.0219] |
| **3-shot Prompt** | | | |
| Rombos vs. Solar-Preview | 0.1767 | <0.0001* | [0.0673, 0.2129] |
| Rombos vs. Phi-4 | 0.1746 | <0.0001* | [0.1239, 0.2407] |
| Rombos vs. Li-14 | -0.0045 | 0.537 | [-0.0521, 0.0604] |
| Rombos vs. Lamarck | 0.0028 | 0.192 | [-0.0148, 0.0559] |
| Solar-Preview vs. Phi-4 | -0.0021 | 0.925 | [-0.0238, 0.1043] |
| Solar-Preview vs. Li-14 | -0.1812 | <0.0001* | [-0.2188, -0.0604] |
| Solar-Preview vs. Lamarck | -0.1739 | <0.0001* | [-0.1958, -0.0567] |
| Phi-4 vs. Li-14 | -0.1791 | <0.0001* | [-0.2515, -0.1132] |
| Phi-4 vs. Lamarck | -0.1718 | <0.0001* | [-0.2232, -0.1128] |
| Li-14 vs. Lamarck | 0.0073 | 0.2355 | [-0.0336, 0.0502] |
| **CoT Prompt** | | | |
| Rombos vs. Solar-Preview | 0.1767 | <0.0001* | [0.1438, 0.2880] |
| Rombos vs. Phi-4 | 0.1101 | <0.0001* | [0.0640, 0.1969] |
| Rombos vs. Li-14 | 0.0848 | <0.0001* | [0.0360, 0.1294] |
| Rombos vs. Lamarck | 0.0805 | 0.0005* | [0.0108, 0.1127] |
| Solar-Preview vs. Phi-4 | -0.0666 | 0.003* | [-0.1600, 0.0034] |
| Solar-Preview vs. Li-14 | -0.0919 | <0.0001* | [-0.1794, -0.0630] |
| Solar-Preview vs. Lamarck | -0.0962 | <0.0001* | [-0.2293, -0.0649] |
| Phi-4 vs. Li-14 | -0.0252 | 0.0425 | [-0.1230, 0.0167] |
| Phi-4 vs. Lamarck | -0.0295 | 0.026 | [-0.1608, 0.0144] |
| Li-14 vs. Lamarck | -0.0043 | 0.291 | [-0.0808, 0.0349] |
| **Adapted Prompt** | | | |
| Rombos vs. Solar-Preview | 0.2028 | <0.0001* | [0.1685, 0.2729] |
| Rombos vs. Phi-4 | 0.2091 | <0.0001* | [0.1470, 0.2766] |
| Rombos vs. Li-14 | 0.0647 | <0.0001* | [0.0276, 0.1444] |
| Rombos vs. Lamarck | 0.0623 | 0.002* | [0.0019, 0.1203] |
| Solar-Preview vs. Phi-4 | 0.0062 | 0.518 | [-0.0685, 0.0475] |
| Solar-Preview vs. Li-14 | -0.1382 | <0.0001* | [-0.1933, -0.0719] |
| Solar-Preview vs. Lamarck | -0.1405 | <0.0001* | [-0.2207, -0.1061] |
| Phi-4 vs. Li-14 | -0.1444 | <0.0001* | [-0.1829, -0.0688] |
| Phi-4 vs. Lamarck | -0.1467 | <0.0001* | [-0.1947, -0.1077] |
| Li-14 vs. Lamarck | -0.0023 | 0.0895 | [-0.0803, 0.0232] |

Supplementary Table 8: Statistical Analysis (Paired Bootstrap Results) for the Micro-averaged Precision. *statistically significant difference at the p < 0.005 accounting for the multiple comparisons problem with (10 pairs per metric).

|  | **Observed Difference** | **p-value** | **99.5% CI** |
| --- | --- | --- | --- |
| **Standard Prompt** | | | |
| Rombos vs. Solar-Preview | 0.0508 | 0.0320 | [-0.0183, 0.1508] |
| Rombos vs. Phi-4 | 0.0396 | 0.1180 | [-0.0275, 0.1019] |
| Rombos vs. Li-14 | 0.0576 | 0.0010* | [0.0151, 0.1016] |
| Rombos vs. Lamarck | 0.0203 | 0.0650 | [-0.0102, 0.0651] |
| Solar-Preview vs. Phi-4 | -0.0112 | 0.3030 | [-0.1168, 0.0383] |
| Solar-Preview vs. Li-14 | 0.0067 | 1.0170 | [-0.0993, 0.0785] |
| Solar-Preview vs. Lamarck | -0.0305 | 0.1840 | [-0.1199, 0.0373] |
| Phi-4 vs. Li-14 | 0.0179 | 0.3290 | [-0.0332, 0.0890] |
| Phi-4 vs. Lamarck | -0.0193 | 0.5260 | [-0.0600, 0.0409] |
| Li-14 vs. Lamarck | -0.0372 | 0.0500 | [-0.0767, 0.0200] |
| **1-shot Prompt** | | | |
| Rombos vs. Solar-Preview | 0.0508 | 0.032 | [-0.0183, 0.1508] |
| Rombos vs. Phi-4 | 0.0396 | 0.118 | [-0.0275, 0.1019] |
| Rombos vs. Li-14 | 0.0576 | 0.001 | [0.0151, 0.1016] |
| Rombos vs. Lamarck | 0.0203 | 0.065 | [-0.0102, 0.0651] |
| Solar-Preview vs. Phi-4 | -0.0112 | 0.303 | [-0.1168, 0.0383] |
| Solar-Preview vs. Li-14 | 0.0067 | 1.017 | [-0.0993, 0.0785] |
| Solar-Preview vs. Lamarck | -0.0305 | 0.184 | [-0.1199, 0.0373] |
| Phi-4 vs. Li-14 | 0.0179 | 0.329 | [-0.0332, 0.0890] |
| Phi-4 vs. Lamarck | -0.0193 | 0.526 | [-0.0600, 0.0409] |
| Li-14 vs. Lamarck | -0.0372 | 0.05 | [-0.0767, 0.0200] |
| **3-shot Prompt** | | | |
| Rombos vs. Solar-Preview | 0.1599 | <0.0001* | [0.0388, 0.1908] |
| Rombos vs. Phi-4 | 0.1742 | <0.0001* | [0.1194, 0.2255] |
| Rombos vs. Li-14 | 0.0164 | 0.068 | [-0.0235, 0.0739] |
| Rombos vs. Lamarck | 0.0061 | 0.1625 | [-0.0168, 0.0562] |
| Solar-Preview vs. Phi-4 | 0.0143 | 0.006 | [-0.0042, 0.1174] |
| Solar-Preview vs. Li-14 | -0.1435 | 0.002* | [-0.1684, -0.0103] |
| Solar-Preview vs. Lamarck | -0.1538 | <0.0001* | [-0.1791, -0.0269] |
| Phi-4 vs. Li-14 | -0.1579 | <0.0001* | [-0.2083, -0.0827] |
| Phi-4 vs. Lamarck | -0.1681 | <0.0001* | [-0.2132, -0.0902] |
| Li-14 vs. Lamarck | -0.0102 | 0.1805 | [-0.0523, 0.0214] |
| **CoT Prompt** | | | |
| Rombos vs. Solar-Preview | 0.1729 | <0.0001* | [0.1409, 0.2799] |
| Rombos vs. Phi-4 | 0.0957 | <0.0001* | [0.0500, 0.1969] |
| Rombos vs. Li-14 | 0.0941 | <0.0001* | [0.0383, 0.1429] |
| Rombos vs. Lamarck | 0.0801 | 0.0005* | [0.0079, 0.1194] |
| Solar-Preview vs. Phi-4 | -0.0773 | 0.0025* | [-0.1591, -0.0051] |
| Solar-Preview vs. Li-14 | -0.0788 | <0.0001* | [-0.1613, -0.0499] |
| Solar-Preview vs. Lamarck | -0.0928 | <0.0001* | [-0.2309, -0.0602] |
| Phi-4 vs. Li-14 | -0.0016 | 0.24 | [-0.1033, 0.0357] |
| Phi-4 vs. Lamarck | -0.0156 | 0.105 | [-0.1580, 0.0325] |
| Li-14 vs. Lamarck | -0.014 | 0.1495 | [-0.0860, 0.0268] |
| **Adapted Prompt** | | | |
| Rombos vs. Solar-Preview | 0.2078 | <0.0001* | [0.1577, 0.2588] |
| Rombos vs. Phi-4 | 0.1852 | <0.0001* | [0.1057, 0.2366] |
| Rombos vs. Li-14 | 0.0699 | <0.0001* | [0.0414, 0.1353] |
| Rombos vs. Lamarck | 0.0592 | 0.0095 | [-0.0082, 0.1071] |
| Solar-Preview vs. Phi-4 | -0.0226 | 0.069 | [-0.0917, 0.0202] |
| Solar-Preview vs. Li-14 | -0.1379 | <0.0001* | [-0.1817, -0.0643] |
| Solar-Preview vs. Lamarck | -0.1486 | <0.0001* | [-0.2246, -0.0998] |
| Phi-4 vs. Li-14 | -0.1153 | <0.0001* | [-0.1457, -0.0291] |
| Phi-4 vs. Lamarck | -0.126 | <0.0001* | [-0.1688, -0.0817] |
| Li-14 vs. Lamarck | -0.0107 | 0.0305 | [-0.0919, 0.0151] |

Supplementary Table 9: Statistical Analysis (Paired Bootstrap Results) for the Micro-averaged F1. *statistically significant difference at the p < 0.005 accounting for the multiple comparisons problem with (10 pairs per metric).

|  | **Observed Difference** | **p-value** | **99.5% CI** |
| --- | --- | --- | --- |
| **Standard Prompt** | | | |
| Rombos vs. Solar-Preview | 0.1129 | <0.0001* | [0.0777, 0.1491] |
| Rombos vs. Phi-4 | 0.1363 | <0.0001* | [0.0950, 0.1740] |
| Rombos vs. Li-14 | 0.0815 | <0.0001* | [0.0539, 0.1059] |
| Rombos vs. Lamarck | 0.0437 | <0.0001* | [0.0247, 0.0634] |
| Solar-Preview vs. Phi-4 | 0.0235 | 0.1060 | [-0.0171, 0.0651] |
| Solar-Preview vs. Li-14 | -0.0313 | 0.0280 | [-0.0741, 0.0092] |
| Solar-Preview vs. Lamarck | -0.0691 | <0.0001* | [-0.1057, -0.0333] |
| Phi-4 vs. Li-14 | -0.0548 | <0.0001* | [-0.0955, -0.0175] |
| Phi-4 vs. Lamarck | -0.0926 | <0.0001* | [-0.1278, -0.0577] |
| Li-14 vs. Lamarck | -0.0378 | <0.0001* | [-0.0612, -0.0108] |
| **1-shot Prompt** | | | |
| Rombos vs. Solar-Preview | 0.2224 | <0.0001* | [0.1665, 0.2765] |
| Rombos vs. Phi-4 | 0.3666 | <0.0001* | [0.3201, 0.4199] |
| Rombos vs. Li-14 | 0.0313 | 0.0020* | [0.0013, 0.0636] |
| Rombos vs. Lamarck | 0.0254 | 0.0015* | [0.0028, 0.0490] |
| Solar-Preview vs. Phi-4 | 0.1442 | <0.0001* | [0.0890, 0.2030] |
| Solar-Preview vs. Li-14 | -0.1911 | <0.0001* | [-0.2497, -0.1392] |
| Solar-Preview vs. Lamarck | -0.1970 | <0.0001* | [-0.2486, -0.1470] |
| Phi-4 vs. Li-14 | -0.3353 | <0.0001* | [-0.3903, -0.2905] |
| Phi-4 vs. Lamarck | -0.3412 | <0.0001* | [-0.3885, -0.2966] |
| Li-14 vs. Lamarck | -0.0059 | 0.2720 | [-0.0322, 0.0199] |
| **3-shot Prompt** | | | |
| Rombos vs. Solar-Preview | -0.0137 | 0.1935 | [-0.0549, 0.0268] |
| Rombos vs. Phi-4 | 0.2485 | <0.0001* | [0.2059, 0.2950] |
| Rombos vs. Li-14 | 0.1194 | <0.0001* | [0.0702, 0.1701] |
| Rombos vs. Lamarck | 0.0176 | 0.0280 | [-0.0059, 0.0431] |
| Solar-Preview vs. Phi-4 | 0.2622 | <0.0001* | [0.2139, 0.3211] |
| Solar-Preview vs. Li-14 | 0.1331 | <0.0001* | [0.0713, 0.2077] |
| Solar-Preview vs. Lamarck | 0.0313 | 0.0230 | [-0.0120, 0.0749] |
| Phi-4 vs. Li-14 | -0.1292 | <0.0001* | [-0.1870, -0.0638] |
| Phi-4 vs. Lamarck | -0.2309 | <0.0001* | [-0.2805, -0.1819] |
| Li-14 vs. Lamarck | -0.1018 | <0.0001* | [-0.1605, -0.0513] |
| **CoT Prompt** | | | |
| Rombos vs. Solar-Preview | 0.2094 | <0.0001* | [0.1718, 0.2466] |
| Rombos vs. Phi-4 | 0.2648 | <0.0001* | [0.2121, 0.3187] |
| Rombos vs. Li-14 | 0.1005 | <0.0001* | [0.0675, 0.1408] |
| Rombos vs. Lamarck | 0.0385 | <0.0001* | [0.0170, 0.0606] |
| Solar-Preview vs. Phi-4 | 0.0554 | 0.0120 | [-0.0097, 0.1241] |
| Solar-Preview vs. Li-14 | -0.1089 | <0.0001* | [-0.1531, -0.0622] |
| Solar-Preview vs. Lamarck | -0.1709 | <0.0001* | [-0.2155, -0.1335] |
| Phi-4 vs. Li-14 | -0.1644 | <0.0001* | [-0.2201, -0.1086] |
| Phi-4 vs. Lamarck | -0.2264 | <0.0001* | [-0.2883, -0.1683] |
| Li-14 vs. Lamarck | -0.0620 | <0.0001* | [-0.1028, -0.0299] |
| **Adapted Prompt** | | | |
| Rombos vs. Solar-Preview | 0.2061 | <0.0001* | [0.1728, 0.2348] |
| Rombos vs. Phi-4 | 0.2029 | <0.0001* | [0.1704, 0.2360] |
| Rombos vs. Li-14 | 0.0789 | <0.0001* | [0.0500, 0.1065] |
| Rombos vs. Lamarck | 0.0372 | <0.0001* | [0.0163, 0.0608] |
| Solar-Preview vs. Phi-4 | -0.0033 | 0.4195 | [-0.0384, 0.0343] |
| Solar-Preview vs. Li-14 | -0.1272 | <0.0001* | [-0.1593, -0.0968] |
| Solar-Preview vs. Lamarck | -0.1689 | <0.0001* | [-0.1987, -0.1395] |
| Phi-4 vs. Li-14 | -0.1239 | <0.0001* | [-0.1552, -0.0913] |
| Phi-4 vs. Lamarck | -0.1657 | <0.0001* | [-0.2006, -0.1308] |
| Li-14 vs. Lamarck | -0.0417 | <0.0001* | [-0.0680, -0.0160] |

Supplementary Table 10: Statistical Analysis (Paired Bootstrap Results) for the Macro-averaged F1. *statistically significant difference at the p < 0.005 accounting for the multiple comparisons problem with (10 pairs per metric).

|  | **Observed Difference** | **p-value** | **99.5% CI** |
| --- | --- | --- | --- |
| **Standard Prompt** | | | |
| Rombos vs. Solar-Preview | 0.0368 | 0.061 | [-0.0225, 0.1449] |
| Rombos vs. Phi-4 | 0.0537 | 0.015 | [-0.0119, 0.1244] |
| Rombos vs. Li-14 | 0.0635 | <0.0001* | [0.0209, 0.1099] |
| Rombos vs. Lamarck | 0.0267 | 0.007 | [-0.0009, 0.0684] |
| Solar-Preview vs. Phi-4 | 0.0169 | 0.977 | [-0.0813, 0.0573] |
| Solar-Preview vs. Li-14 | 0.0267 | 0.697 | [-0.0772, 0.0926] |
| Solar-Preview vs. Lamarck | -0.0101 | 0.464 | [-0.1046, 0.0471] |
| Phi-4 vs. Li-14 | 0.0098 | 0.527 | [-0.0415, 0.0783] |
| Phi-4 vs. Lamarck | -0.027 | 0.202 | [-0.0708, 0.0283] |
| Li-14 vs. Lamarck | -0.0368 | 0.051 | [-0.0789, 0.0178] |
| **1-shot Prompt** | | | |
| Rombos vs. Solar-Preview | 0.1554 | <0.0001* | [0.0532, 0.2367] |
| Rombos vs. Phi-4 | 0.1972 | <0.0001* | [0.1576, 0.2712] |
| Rombos vs. Li-14 | 0.0202 | 0.22 | [-0.0424, 0.0706] |
| Rombos vs. Lamarck | -0.0414 | 0.0705 | [-0.0618, 0.0240] |
| Solar-Preview vs. Phi-4 | 0.0417 | 0.008 | [-0.0088, 0.1510] |
| Solar-Preview vs. Li-14 | -0.1352 | <0.0001* | [-0.1941, -0.0533] |
| Solar-Preview vs. Lamarck | -0.1968 | <0.0001* | [-0.2459, -0.0756] |
| Phi-4 vs. Li-14 | -0.1769 | <0.0001* | [-0.2573, -0.1437] |
| Phi-4 vs. Lamarck | -0.2386 | <0.0001* | [-0.2972, -0.1797] |
| Li-14 vs. Lamarck | -0.0616 | 0.083 | [-0.0907, 0.0236] |
| **3-shot Prompt** | | | |
| Rombos vs. Solar-Preview | 0.145 | <0.0001* | [0.0383, 0.1811] |
| Rombos vs. Phi-4 | 0.197 | <0.0001* | [0.1509, 0.2531] |
| Rombos vs. Li-14 | 0.0226 | 0.038 | [-0.0214, 0.0874] |
| Rombos vs. Lamarck | 0.0099 | 0.0795 | [-0.0125, 0.0610] |
| Solar-Preview vs. Phi-4 | 0.052 | <0.0001* | [0.0329, 0.1547] |
| Solar-Preview vs. Li-14 | -0.1224 | 0.006 | [-0.1582, 0.0082] |
| Solar-Preview vs. Lamarck | -0.1351 | <0.0001* | [-0.1632, -0.0190] |
| Phi-4 vs. Li-14 | -0.1744 | <0.0001* | [-0.2282, -0.1022] |
| Phi-4 vs. Lamarck | -0.1871 | <0.0001* | [-0.2353, -0.1277] |
| Li-14 vs. Lamarck | -0.0127 | 0.1535 | [-0.0547, 0.0243] |
| **CoT Prompt** | | | |
| Rombos vs. Solar-Preview | 0.1948 | <0.0001* | [0.1573, 0.2974] |
| Rombos vs. Phi-4 | 0.1387 | <0.0001* | [0.0908, 0.2382] |
| Rombos vs. Li-14 | 0.101 | <0.0001* | [0.0513, 0.1506] |
| Rombos vs. Lamarck | 0.084 | 0.0005* | [0.0173, 0.1230] |
| Solar-Preview vs. Phi-4 | -0.0561 | 0.0135 | [-0.1312, 0.0150] |
| Solar-Preview vs. Li-14 | -0.0938 | <0.0001* | [-0.1729, -0.0612] |
| Solar-Preview vs. Lamarck | -0.1108 | <0.0001* | [-0.2440, -0.0755] |
| Phi-4 vs. Li-14 | -0.0377 | 0.008 | [-0.1427, 0.0053] |
| Phi-4 vs. Lamarck | -0.0547 | 0.0025* | [-0.2046, -0.0021] |
| Li-14 vs. Lamarck | -0.017 | 0.092 | [-0.0922, 0.0177] |
| **Adapted Prompt** | | | |
| Rombos vs. Solar-Preview | 0.2131 | <0.0001 | [0.1702, 0.2732] |
| Rombos vs. Phi-4 | 0.2094 | <0.0001 | [0.1366, 0.2633] |
| Rombos vs. Li-14 | 0.0803 | <0.0001 | [0.0475, 0.1473] |
| Rombos vs. Lamarck | 0.0661 | 0.0025 | [0.0008, 0.1179] |
| Solar-Preview vs. Phi-4 | -0.0037 | 0.3075 | [-0.0723, 0.0306] |
| Solar-Preview vs. Li-14 | -0.1328 | <0.0001 | [-0.1814, -0.0657] |
| Solar-Preview vs. Lamarck | -0.147 | <0.0001 | [-0.2240, -0.1118] |
| Phi-4 vs. Li-14 | -0.1291 | <0.0001 | [-0.1667, -0.0521] |
| Phi-4 vs. Lamarck | -0.1433 | <0.0001 | [-0.1875, -0.1013] |
| Li-14 vs. Lamarck | -0.0142 | 0.0215 | [-0.0914, 0.0126] |

Supplementary Table 11: Statistical Analysis (Paired Bootstrap Results) for the Overall Accuracy on the CDE “Previously conducted mammography mentioned?” using the standard and the adapted prompt. *statistically significant difference at the p < 0.005 accounting for the multiple comparisons problem with (10 pairs per metric).

|  | **Observed Difference** | **p-value** | **99.5% CI** |
| --- | --- | --- | --- |
| **Standard Prompt** |  |  |  |
| Rombos vs. Solar-Preview | 0.1639 | 0.0230 | [-0.0164, 0.3607] |
| Rombos vs. Phi-4 | 0.1311 | 0.0240 | [-0.0164, 0.2951] |
| Rombos vs. Li-14 | 0.1475 | <0.0001* | [0.0328, 0.2787] |
| Rombos vs. Lamarck | 0.0492 | 0.2300 | [-0.0492, 0.1476] |
| Solar-Preview vs. Phi-4 | -0.0328 | 0.6770 | [-0.2131, 0.1311] |
| Solar-Preview vs. Li-14 | -0.0164 | 0.8780 | [-0.2131, 0.1803] |
| Solar-Preview vs. Lamarck | -0.1148 | 0.1030 | [-0.2951, 0.0656] |
| Phi-4 vs. Li-14 | 0.0164 | 0.7890 | [-0.0656, 0.0984] |
| Phi-4 vs. Lamarck | -0.0820 | 0.0750 | [-0.2131, 0.0328] |
| Li-14 vs. Lamarck | -0.0984 | 0.0040* | [-0.2131, -0.0164] |
| **Adapted Prompt** |  |  |  |
| Rombos vs. Solar-Preview | 0.3279 | <0.0001* | [0.1311, 0.5246] |
| Rombos vs. Phi-4 | 0.0328 | 0.6100 | [-0.0984, 0.1803] |
| Rombos vs. Li-14 | -0.0164 | 0.7770 | [-0.0984, 0.0656] |
| Rombos vs. Lamarck | -0.0164 | 0.7770 | [-0.0984, 0.0656] |
| Solar-Preview vs. Phi-4 | -0.2951 | <0.0001* | [-0.4590, -0.1639] |
| Solar-Preview vs. Li-14 | -0.3443 | <0.0001* | [-0.5246, -0.1803] |
| Solar-Preview vs. Lamarck | -0.3443 | <0.0001* | [-0.5246, -0.1803] |
| Phi-4 vs. Li-14 | -0.0492 | 0.2240 | [-0.1639, 0.0328] |
| Phi-4 vs. Lamarck | -0.0492 | 0.2240 | [-0.1639, 0.0328] |
| Li-14 vs. Lamarck | 0.0000 | 2.0000 | [0.0000, 0.0000] |

Supplementary Table 12: Required time in seconds to execute the script on the entire dataset of 61 reports as well as the time needed per report, depending on the prompting techniques and the LLM used.

|  | **Default Prompt** | **1-Shot** | **3-Shot** | **CoT** | **Adapted Prompt** |
| --- | --- | --- | --- | --- | --- |
| **Rombos** | 2665.69; 43.70 | 4988.69; 81.78 | 10549.91, 172.95 | 4773.24; 78.25 | 2614.33; 42.86 |
| **Solar Preview** | 6581.68; 107.90 | 12026.53; 197.16 | 26037.45; 426.84 | 11739.22; 192.45 | 6491.45; 106.42 |
| **Phi-4** | 2489.52; 40.81 | 4664.31; 76.46 | 10232.56; 167.75 | 4137.54; 67.83 | 2423.45; 39.73 |
| **Li** | 2625.25; 43.04 | 4929.28; 80.81 | 10568.65; 173.26 | 4812.55; 78.89 | 2607.59; 42.75 |
| **Lamarck** | 2746.58; 45.03 | 5219.44; 85.56 | 11159.29; 182.94 | 5118.34; 83.91 | 2718.58; 44.57 |
